# Clickable Photolabile Linkers for Convenient Immobilization and Light-Controlled Release of Substrates from Solid Support

**DOI:** 10.1021/jacs.6c18307

**Published:** 2026-10-14

**Authors:** Selvanathan Arumugam, Nannan Lin, Chen Zhao, Jin Xie, Vladimir V. Popik

**Affiliations:** Department of Chemistry, 1355 University of Georgia, Athens, Georgia 30602, United States

## Abstract

Photolabile linkers (PLLs) enable spatiotemporal control over substrate release but often suffer from slow photorelease kinetics, low quantum yields, or limited functionalization options. Here, we report a family of heterobifunctional PLLs based on (3-hydroxy-5-alkoxynaphthalen-2-yl)­methyl (NQMP) chromophores that combine rapid, high-yielding photochemical cleavage with orthogonal conjugation chemistries for efficient substrate immobilization and light-triggered release. Six heterobifunctional NQMP-based linkers bearing terminal acetylene, cyclooctyne, amine, carboxyl, allyl, and biotin groups were synthesized and employed for the immobilization of fluorescent dyes, acetylsalicylic acid, biotin, avidin, and doxorubicin on silica microspheres and mesoporous silica nanoparticles. Mild UV-A irradiation resulted in efficient release of the attached payloads while preserving the integrity of the solid support. Mesoporous silica nanoparticles provide high loading capacity and are suitable for light-controlled drug delivery. The modular design, excellent photochemical efficiency, orthogonal conjugation chemistries, and compatibility with diverse substrates make NQMP-based PLLs versatile tools for reversible surface functionalization, controlled cargo release, affinity capture, and photoresponsive drug delivery applications.

## Graphical abstract

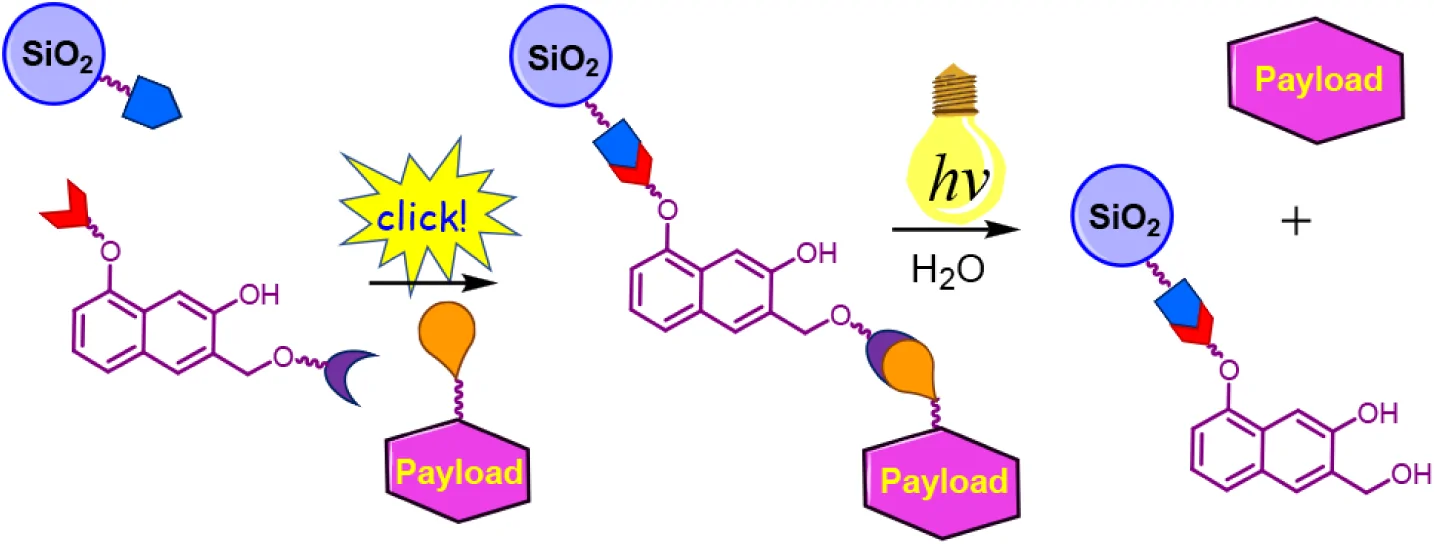

## Introduction

Photolabile linkers (PLLs) allow for the spatiotemporal control of the release of active substrates from a solid or polymeric support, as well as the cleavage of the bond between two substrates. Many light-directed drug delivery systems^1^ are based on the use of a photolabile linker to connect a bioactive molecule to a targeting moiety^2^ or a nanocarrier.^3^ Alternatively, a drug can be loaded into a PLL-based phototriggered delivery vehicle,^4^ such as polymer,^5^ hydrogel,^6^ micelle,^7^ liposome, polymersome,^8^ or nanocage.^9^ PLLs are also employed for surface and polymer photopatterning,^10^ as well as for the design of photodegradable polymers.^11^ Some super-resolution fluorescence microscopy techniques rely on the spatial control of bond cleavage enabled by photolabile linkers.^12^ In addition, the use of PLLs allows for the reagentless release of immobilized substrates, e.g., in solid-support peptide and oligosaccharide syntheses^13^ or in “pull down” procedures for protein enrichment.^14^ The design of the vast majority of PLLs is based on the photohydrolysis of o-nitrobenzyl, coumarin-4-ylmethyl, or BODIPY-8-methyl esters.^15,16^ While an o-nitrobenzyl linker usually offers a good quantum yield and strong absorbance in the UVA region, the substrate release is often slow, as this reaction proceeds via several dark steps.^15^ The photocleavage of coumarin-4-ylmethyl and BODIPY-8-methyl esters is much faster but suffers from a relatively low quantum yield.

An ideal photolabile linker should be stable in the dark but undergo efficient cleavage with high quantum and chemical yields upon irradiation. A short lifetime of the activated linker is crucial for kinetic and time-resolved studies, while substantial absorbance above 300 nm is beneficial for biochemical applications. For biological applications, linker design should avoid hydrolytically and enzymatically labile bonds. Finally, the photolabile linker should be equipped with convenient handles for easy attachment of the payload and the substrate.

Here, we report the straightforward synthesis and discuss the reactivity of PLLs based on (3-hydroxy-5-alkoxynaphth-2-yl)­methyl derivatives, dubbed **NQMP**s for **o**-**N**aphtho**Q**uinone **M**ethide **P**recursors (*vide infra*). We have previously shown that the light-induced carbon-heteroatom bond cleavage in NQMP esters, ethers, amines, and thioethers occurs within 12 μs upon excitation to produce the reactive intermediate 2,3-napthoquinone-3-methide (**oNQM**) (Scheme 1).^17^ In aqueous solution, the initially formed oNQM undergoes rapid (τ = 7.4 ms) hydration to produce 3-hydroxy-2-naphthalenemethanol (**NQMP-OH**, Scheme 1).^17^ The photoheterolysis of the C-X bond in NQMP derivatives is characterized by excellent chemical yields (above 90%) and quantum yields (Φ = 30–60%).^17,18^ The cleavage efficiency of these linkers does not depend on the nature of the leaving group.^17a,18b^ Even a fluorescent reporter attached to an NQMP PLL does not affect the quantum efficiency of the photocleavage (Φ = 38–42%; NQMP-OR’, R= Dansyl, Scheme 1).^17d^

**Scheme 1:**
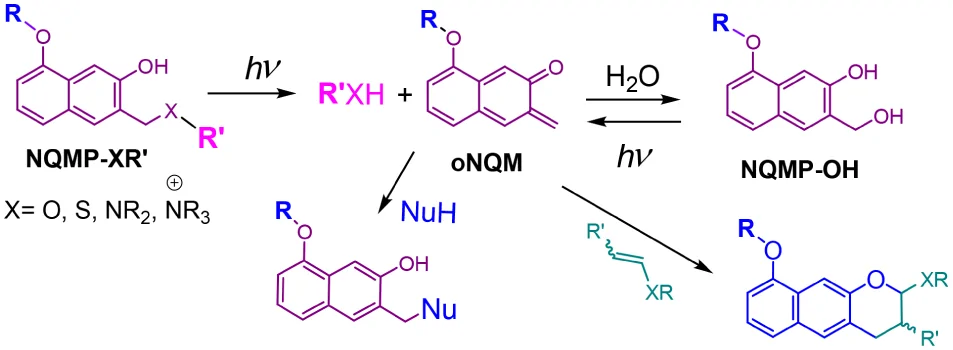
Photochemistry of (3-Hydroxy-5-alkoxynaphthalen-2-yl)­methyl (NQMP) PLLs

The oNQM intermediate can be intercepted by a very fast Michael^17b^ reaction (e.g., *k*_RSH_ = 10^5^– 10^8^ M^–1^ s^–1^) or a facile IEDDA addition to electron-rich alkenes (*k* = 10^4^–10^5^ M^–1^ s^–1^).^17b,19^

The present report focuses on the use of NQMP PLLs for the efficient immobilization or encapsulation, and the subsequent photorelease, of various compounds from a solid support. In this approach, a surface or a substrate (**S1**) is attached to the 5-position of **the NQMP** core via a photostable ether linkage, while the second substrate (**S2**) is connected via a photolabile benzylic ether or ester bond (**1**, Scheme 2). Upon UVA irradiation in aqueous solutions, photohydrolysis of the linker results in the release of substrate **S2** as an alcohol or carboxylate, while substrate **S1** remains connected to NQMP-OH (**2**). In a nonhydroxylic solvent, the oNQM intermediate can be intercepted by Michael^17^ or IEDDA reactions.^17a,19^

**Scheme 2:**
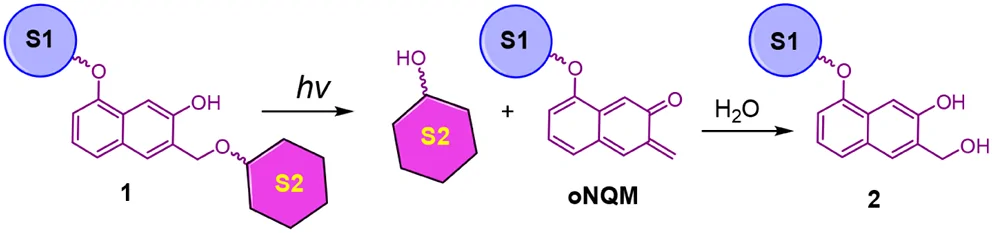
Substrate Photo-Release Using NQMP-based PLL

Product **2** can be further derivatized using photo-Michael or photo-Diels–Alder chemistry, if needed.^17,18^

## Results and Discussion

We have synthesized and tested the utility of six NQMP-based PLLs (**3a**–**f**) equipped with various functionalities for the efficient and high-yield immobilization of substrates ranging from small molecules to proteins (Scheme 3).

**Scheme 3:**
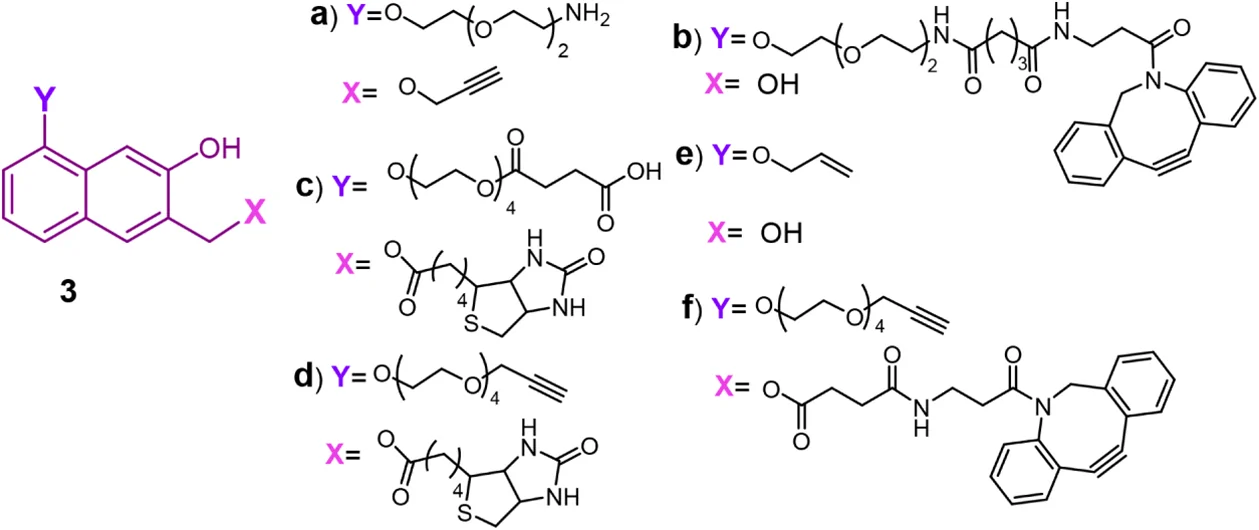
NQMP-Based Photolabile Linkers

To achieve efficient and high-yield immobilization, linkers possess heterobifunctional termini with orthogonal reactivity: the CuAAC click reaction and nucleophilic substitution/addition (**3a**), the SPAAC click reaction and esterification (**3b**), ester/amide formation and biotin–avidin binding (**3c**), the CuAAC click reaction and biotin–avidin binding (**3d**), electrophilic addition and esterification (**3e**), and CuAAC and SPAAC click reactions (**3f**; Scheme 3).

### Immobilization and Photorelease of Substrates from a Solid Support

Two types of substrates have been explored for the immobilization and photorelease of payload molecules using PLLs **3**: carboxyl-functionalized silica microbeads (∼1 μm) and mesoporous silica nanoparticles (50–100 nm).^20^ Silica has been selected for its low toxicity and chemical stability. Fluorescent dyes (Texas Red and fluorescein), a small drug molecule (aspirin), a large drug molecule (doxorubicin), a natural bioactive compound (*D*-biotin, i.e., vitamin B7), and a protein (avidin) were selected as payload molecules for these feasibility studies.

### Photorelease of Fluorescent Dyes from Silica Microbeads

The carboxyl-functionalized silica microbeads (SiMB-CO_2_H) were derivatized with PLL **3a** by EDC/DMAP-assisted coupling in dry DMF (Scheme 4).^20^ In order to achieve complete conversion of all carboxylic groups on the silica surface,^21^ a 10-fold molar excess of **3a** was employed, and the reaction was allowed to proceed for 48 h. The derivatized beads were washed with DMF multiple times until the wash contained no UV-detectable amount of **3a**. The payload, a fluorescent dye (Texas Red azide **4**), was attached to the propargyl terminus of PLL **3a** using CuAAC click chemistry to produce fluorescent dye-derivatized silica beads (**Si-NQMP-TR**, Scheme 4).^20^ The DMF-washing procedure with UV control was repeated to remove all traces of unbound dye.

**Scheme 4:**
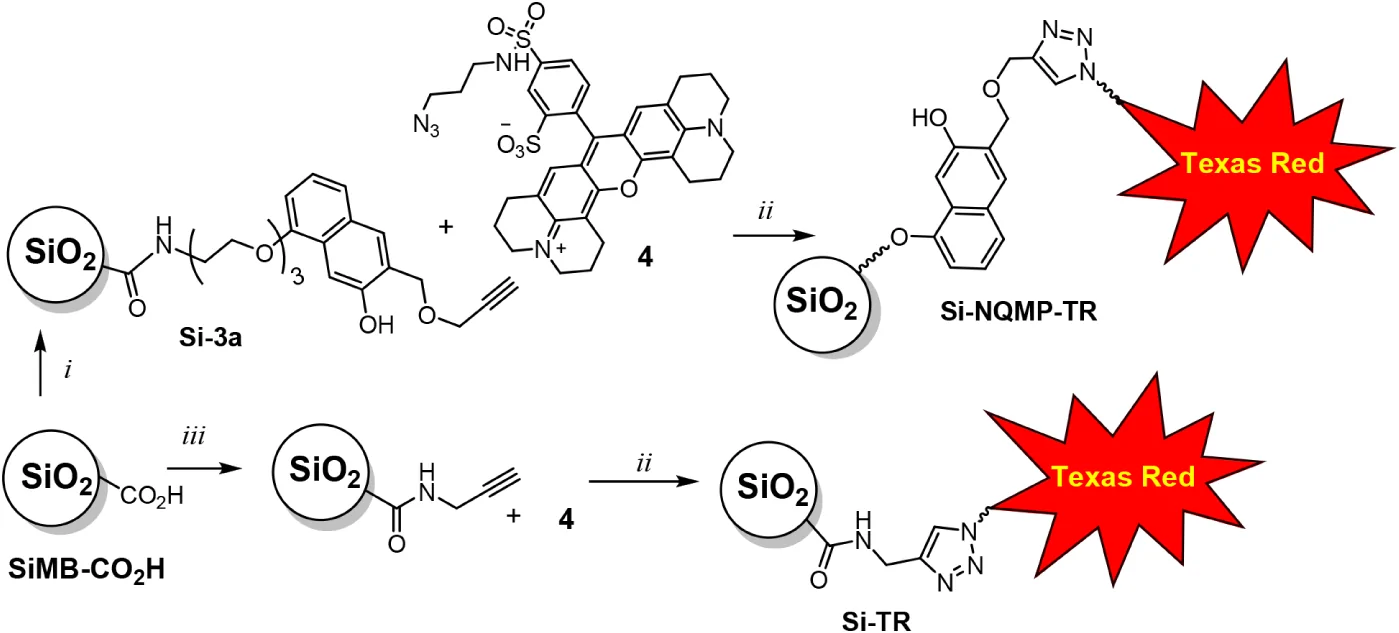
Immobilization of Texas Red on the Silica Microbeads via Photolabile (**Si-NQMP-TR**) or Photostable Linker (**Si-TR**)­^a^

a Reagents and conditions: (i) NH_2_-EG_3_-NQMP-propargyl (**3a**), EDC, DMAP; (ii) CuI, PMDETA; (iii) propargyl amine, EDC, DMAP, PMDETA.

Texas Red fluorescent dye was also immobilized on SiMB-CO_2_H via a photostable linker to serve as a control. Thus, carboxyl groups on the surface of the microbeads were converted into terminal acetylene moieties via EDC/DMAP-mediated coupling with propargylamine. Subsequent CuAAC click reaction with **4** produced Texas Red-coated SiMBs (**Si-TR**, Scheme 4).^20^

The fluorescent dye–derivatized silica beads (**Si-NQMP-TR**) were spin-coated on a glass slide from an acetonitrile suspension using two concentrations (2 g L^–1^ and 0.02 g L^–1^) and the images were recorded using a fluorescence microscope with excitation at 567 nm. Lumps of silica particles exhibit bright fluorescence (Figure S1).^20^ Next, the fluorescent **Si-NQMP-TR** beads were suspended in aqueous acetonitrile (ca 1 g L^–1^) and irradiated under vigorous stirring using 300 or 350 nm fluorescent lamps. The particles were separated by centrifugation, washed with DMF, and spin-coated on a glass slide (at 1 g L^–1^). The images of the resulting slides were acquired with a microscope both in transmission mode (as a loading control) and in fluorescence mode with excitation at 568 nm (Figure 1). The beads irradiated with 300 nm lamps for 2 min show a complete loss of fluorescence, indicating efficient cleavage of NQMP linker **3a** and release of the payload dye (Scheme 5). The complete release of the dye from **Si-NQMP-TR** under 350 nm irradiation required 5 min of exposure. The slower cleavage of PLL **3a** with 350 nm light is apparently due to the lower extinction coefficient of the NQMP chromophore at this wavelength (the UV spectrum of NQMP-biotin is presented in Figure S5).

**Figure 1:**
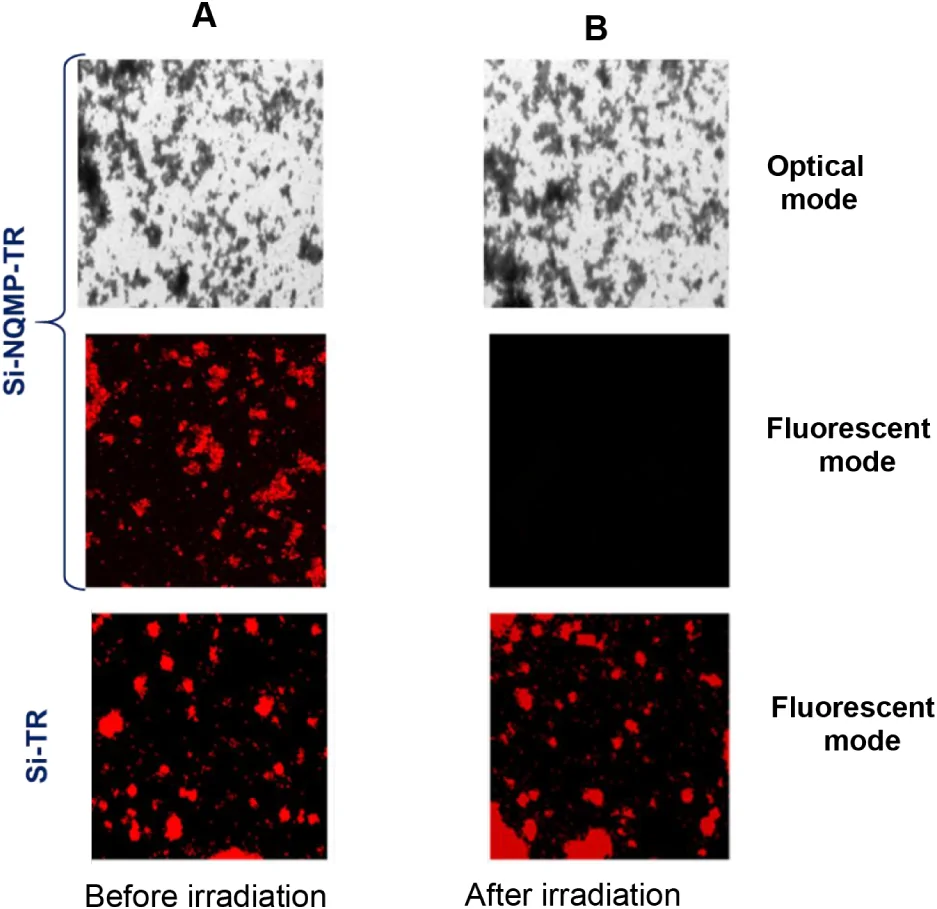
Microscopic images of the Texas Red-derivatized silica microbeads (**Si-NQMP-TR**) spin-coated on a glass slide before (panel **A**) and after (panel **B**) irradiation (2 min at 300 nm). The upper images are recorded in the transmission mode, while the lower images are recorded in the fluorescence mode with 568 nm excitation.

**Scheme 5:**
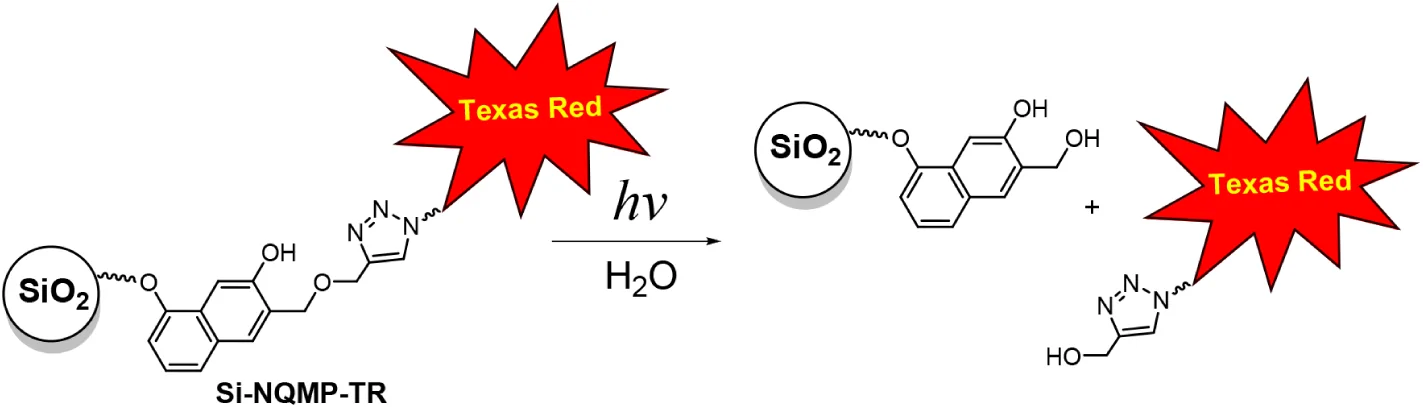
Photorelease of Texas Red Dye from the Silica Microbeads **Si-NQMP-TR**

Lower two panels show fluorescent images of the **Si-TR** control beads (Scheme 4) before (panel **A**) and after 10 min of irradiation at 300 nm (panel **B**).

The loss of emission from the Texas Red-derivatized particles might be caused by photobleaching of the dye under UV irradiation. To account for this effect, the microbeads with the dye immobilized via a photostable linker (**Si-TR**, Scheme 4) were irradiated with 300-nm lamps in vortexed aqueous acetonitrile suspension (ca 1 g L^–1^) for 10 min; the beads were separated by centrifugation and washed with DMF. As illustrated in Figure 1 (two lower panels), the silica beads remained brightly fluorescent after irradiation. The supernatant of the irradiated suspension showed no detectable fluorescence or UV absorbance attributable to the dye. This experiment demonstrated that the dye is not released from the beads in the absence of the NQMP linker, and that it is relatively stable under the given irradiation conditions.

The amount of dye immobilized and photochemically released from the silica beads was determined by measuring the concentration of the dye in the supernatant liquid of the irradiated **Si-NQMP-TR** particles. Five milliliters of a vortexed suspension of **Si-NQMP-TR** in aqueous acetonitrile (1 g L^–1^) were irradiated for 2 min with 300-nm lamps, and the particles were separated by centrifugation and washed with DMF. While the washes of the unexposed particles contained no detectable dye, the supernatant of the silica bead dispersion after photolysis was brightly fluorescent. The concentration of the dye in the combined washes (10 mL) was ∼4.6 μM, as determined by comparing the integrated emission intensity with the calibration curve (Figure 2).^20^ The amount of released dye (0.046 μmol) represents 92% of the theoretical payload density (5 mg at 0.01 μmol per mg of silica beads).^21^

**Figure 2:**
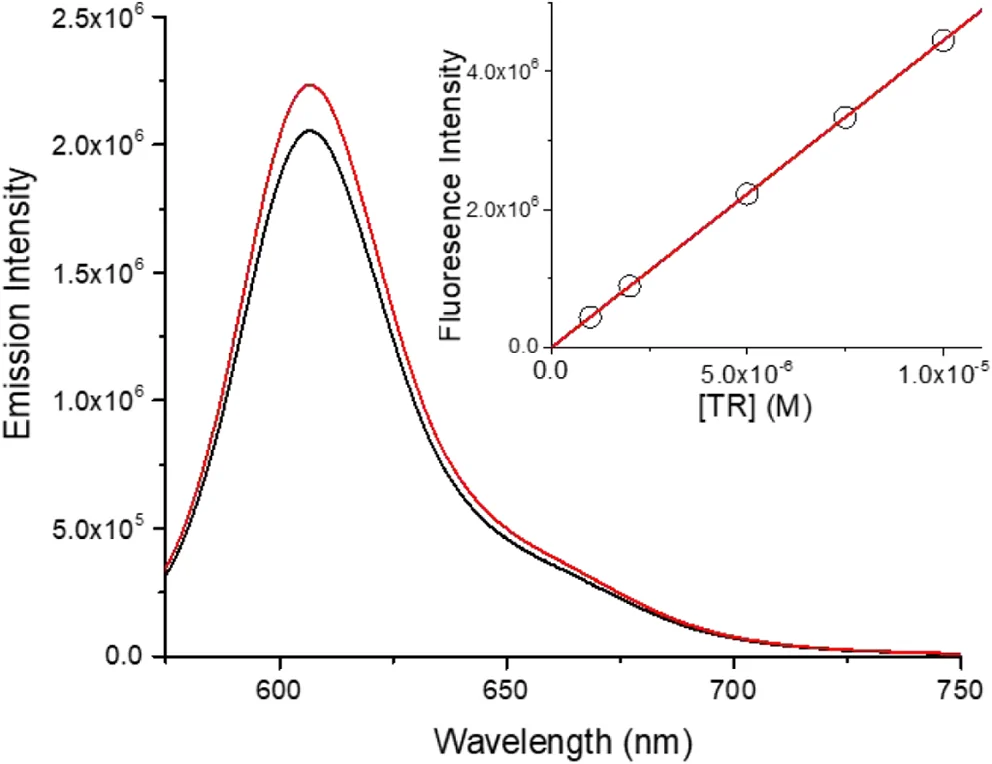
Fluorescence spectra of the combined supernatant and washes after the irradiation of **Si-NQMP-TR** fluorescent beads (black line) and of a 5.0 μM solution of Texas Red in CH_3_CN:DMF (1:1, red line). The inset shows the calibration curve of Texas Red in CH_3_CN:DMF.

The alternative immobilization–photorelease strategy, where attachment of the payload molecule is achieved via SPAAC click chemistry and photocleavage occurs at the covalent bond between the solid support and the linker, has been explored. This approach, realized with the use of PLL **3b**, allows for the reuse of the solid support particles, if needed. The carboxyl groups on the surface of SiMBs were converted into activated NHS ester moieties (**SiMB-NHS**) by treatment with *N*-hydroxysuccinimide, EDC, and DMAP (Scheme 6).^20^ The resulting beads were incubated for 2 days with a 10-fold excess of linker **3b** in DMF to give **Si-NQMP-ADIBO**. The beads were repeatedly washed with DMF until no **3b** could be detected in the supernatant by UV spectroscopy. 5-fold excess of azido-fluorescein was added to a stirred suspension of the beads in dry DMF (10 mg/mL), and the mixture was incubated for 2 h to produce SiMBs derivatized with fluorescein via PLL **3b** (**Si-NQMP-Fluor**, Scheme 6).

**Scheme 6:**
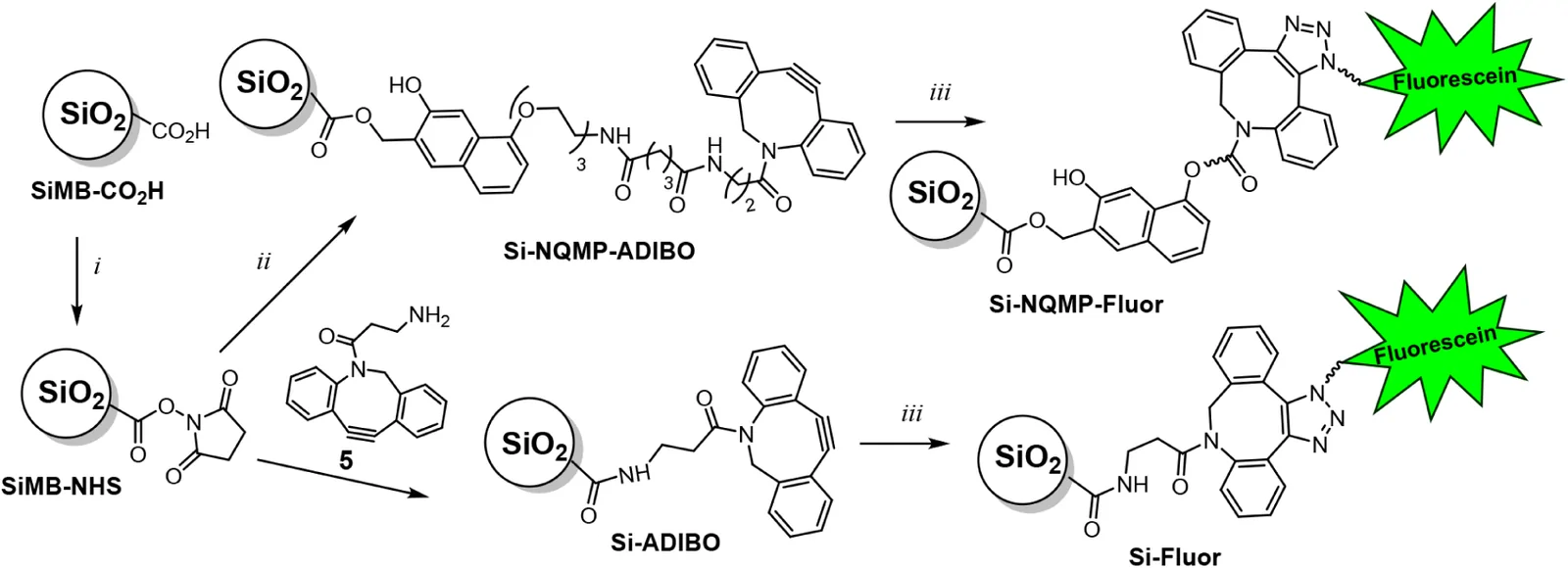
Immobilization of Fluorescein on Silica Microbeads via Photolabile (**Si-NQMP-Fluor**) or Photostable (**Si-Fluor**) Linker^a^

a Reagents and conditions: (i) NHS, EDC, DMAP, DMF; (ii) 3b, Et_3_N, THF; (iii) azido-fluorescein, DMF.

The control beads, with a photostable linker between SiMBs and the dye, were prepared by treating **Si-NHS** with ADIBO-amine (**5**)^22^ in DMF, yielding ADIBO-derivatized beads (**Si-ADIBO**), followed by washing and incubation with a fivefold excess of azido-fluorescein for 2 h. The resulting fluorescein-derivatized SiMBs via a photostable linker (**Si-Fluor**) were thoroughly washed to remove all traces of remaining reagents (Scheme 6).

The fluorescein-derivatized beads (**Si-NQMP-Fluor** and **Si-Fluor**) were spin-coated on glass slides from an aqueous acetonitrile suspension (1 g L^–1^) and the images were recorded with a fluorescence microscope using a 488 nm excitation wavelength (Figure 3, Panel **A**). Lumps of silica particles exhibit bright fluorescence in the green channel. The fluorescent beads (**Si-NQMP-Fluor**) were then suspended in acetonitrile (1 g L^–1^) and irradiated with 300 nm lamps for 2 min. The particles were separated by centrifugation, washed with DMF, and spin-coated on a glass slide (at 1 g L^–1^). The images of the resulting slides were acquired by a microscope in the transmission mode (as a load control) and in the fluorescence mode with 488 nm excitation (Figure 3, Panel **B**). The irradiated **Si-NQMP-Fluor** beads show complete loss of fluorescence, indicating efficient cleavage of NQMP linker **3b** and release of the dye (Scheme 7). As in the case of **Si-NQMP-TR**, the complete release of fluorescein under irradiation with the same number of 350 nm lamps required 5 min of exposure.

**Figure 3:**
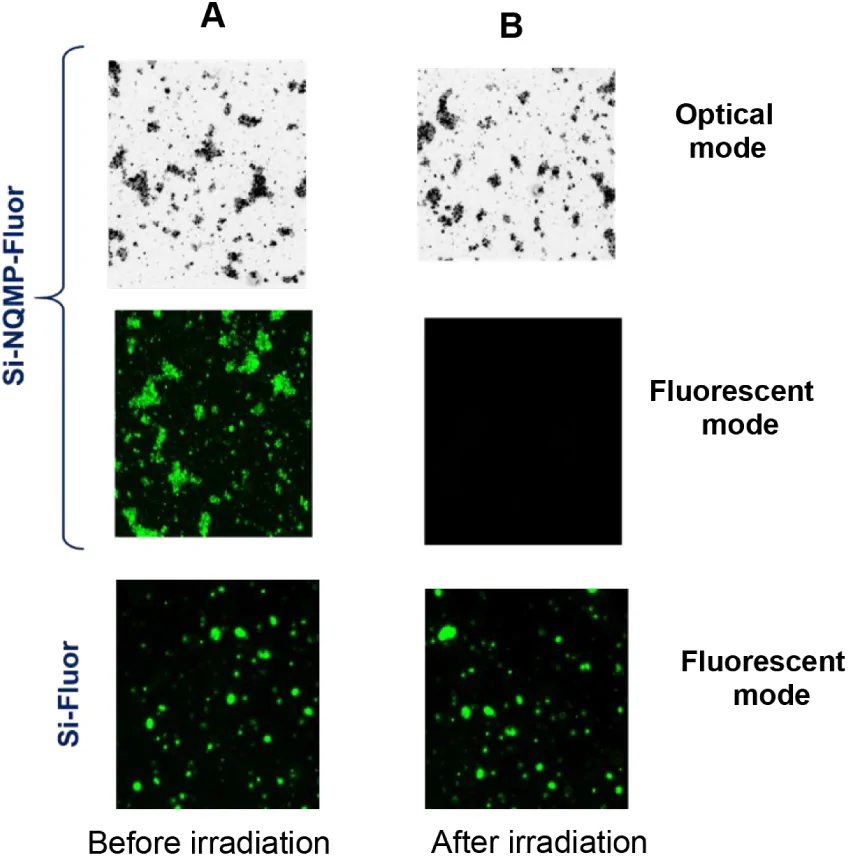
Microscope images of the fluorescein-derivatized silica microbeads (**Si-NQMP-Fluor**) spin-coated on a glass slide before (panel **A**) and after (panel **B**) irradiation (2 min at 300 nm). The upper images are recorded in the transmission mode, while the lower images are recorded in the fluorescence mode with 488 nm excitation.

**Scheme 7:**
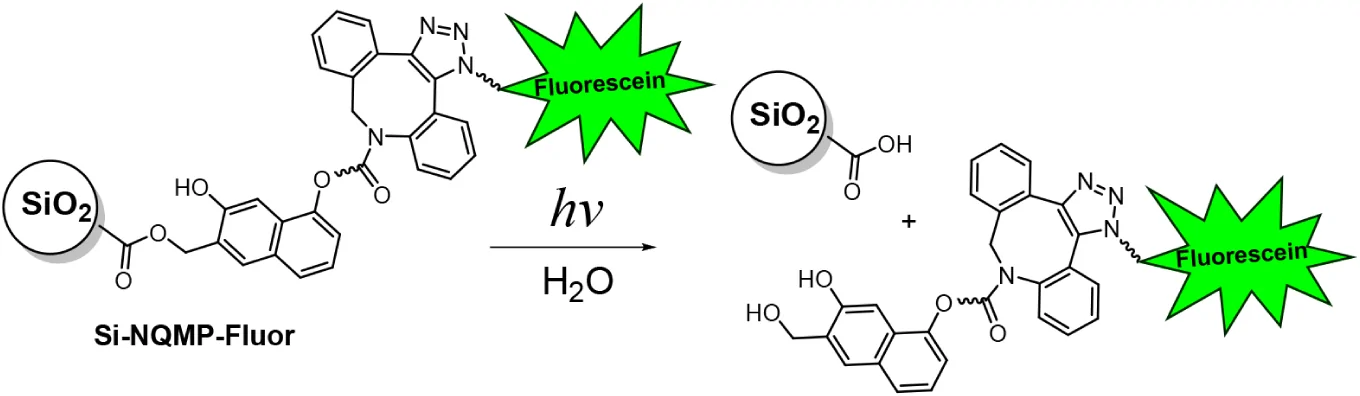
Photorelease of Fluorescein from the Silica Microbeads **Si-NQMP-Fluor** ^a^

a The lower two panels show fluorescent images of the **Si-Fluor** control beads (Scheme 6) before (panel **A**) and after 10 min of irradiation at 300 nm (panel **B**).

A stirred suspension of the photostable, fluorescein-derivatized silica microbeads (**Si-Fluor**) in aqueous acetonitrile was exposed to 300-nm light for 10 min, separated by centrifugation, and washed with DMF. The resulting microbeads still showed fluorescein emission (Figure 3, lower panels). No dye was detected in the supernatant by fluorescence or UV spectroscopy. This observation indicates that the dye is not released in the absence of the photolabile linker, and there is no significant photobleaching of immobilized fluorescein during photolysis.

Quantification of the released dye from **Si-NQMP-Fluor** was conducted using the procedure described above for **Si-NQMP-TR**. A stirred suspension of 5 mg of **Si-NQMP-Fluor** beads in 5 mL of aqueous acetonitrile was irradiated for 2 min with 300 nm lamps, and the particles were separated by centrifugation and washed with DMF (5 mL in total). The concentration of the dye in the combined washes (10 mL) was ∼4.65 μM, as determined by comparing the integrated emission intensity with the calibration curve (Figure 4).^20^ The amount of released dye (0.0465 μmol) constitutes 93% of the theoretical payload density (5 mg of 0.01 μmol/mg of silica beads).^21^

**Figure 4:**
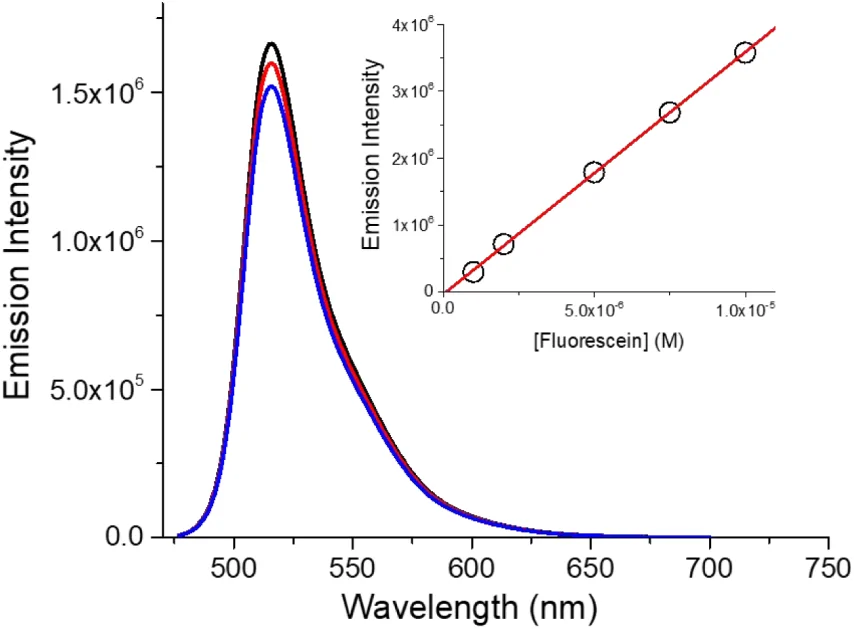
Fluorescence spectra of the combined supernatant and washes after the irradiation of **Si-NQMP-Fluor** fluorescent beads (red line), a 5.0 μM solution of fluorescein azide in CH_3_CN:DMF (1:1, black line), and fluorescein released from reused silica microbeads. The inset shows the calibration curve of fluorescein azide in CH_3_CN:DMF.

Since the silica beads are restored to their native form (SiMB-CO_2_H) after the photo-triggered release of a payload, we have explored the possibility of reusing the silica particles. The SiMB-CO_2_H particles isolated from the fluorescein quantification experiment were submitted to the derivatization procedure outlined above (Scheme 6) to produce **Si-NQMP-Fluor**. This time, a suspension of microbeads in aqueous acetonitrile was irradiated using 350-nm lamps for 5 min. The integral intensity of the fluorescent band at 520 nm in the emission spectrum of the combined supernatant and DFM washes (10 mL) corresponds to 4.46 μM of fluorescein, which represents approximately 96% of the dye concentration released from the functionalized silica when used for the first time. This observation indicates that the catch-and-release process does not significantly impact the functional density of the silica particles.

### Photorelease of Acetylsalicylic Acid (ASA) from Mesoporous Silica Nanoparticles (MSNPs)

Mesoporous silica nanoparticles (MSNPs) possess a much larger surface area than silica microbeads and allow for both immobilization and encapsulation of payload molecules. In this work, MSNPs were prepared using a sol–gel process, followed by acid treatment.^20,23^ MSNPs were then derivatized with the acetylsalicylic acid (ASA)-bearing photolabile linker **3e**-ASA ester (**6**) via triethoxysilane coupling to surface silanols (Scheme 8). First, PLL **3e** was converted to **3e**-ASA ester (**6**) using EDC/DMAP conditions. NQMP-ASA derivatization of the surface and pores of MSNPs was achieved by the catalytic hydrosilylation of **6** with triethoxysilane in the presence of MSNPs (Scheme 8).^20^

**Scheme 8:**
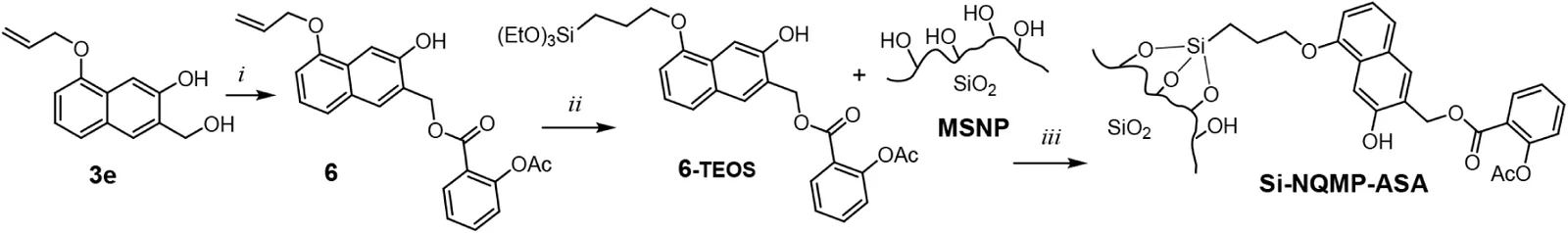
Immobilization of Acetylsalicylic Acid on Mesoporous Silica Nanoparticles Using allyl-NQMP Photolabile Linker **3e** ^a^

a Reagents and conditions: (i) ASA, EDC, DMAP, DMF, 39%; (ii) (EtO)_3_SiH, Karstedt’s catalyst, 80%; (iii) toluene, reflux.

The TEM images of the original MSNs (Figure 5, panel **A**) and acetylsalicylic acid-derivatized MSNPs (Figure 5, panel **B**) show no significant morphological change in the MSNPs upon derivatization. On the other hand, the ζ-potential of the MSNs changed from −0.84 to 28.5 mV during the conversion from silica particles to **Si-NQMP-ASA**, supporting the conversion of the majority of hydroxyl groups on the silica surface into aromatic ester moieties.

**Figure 5:**
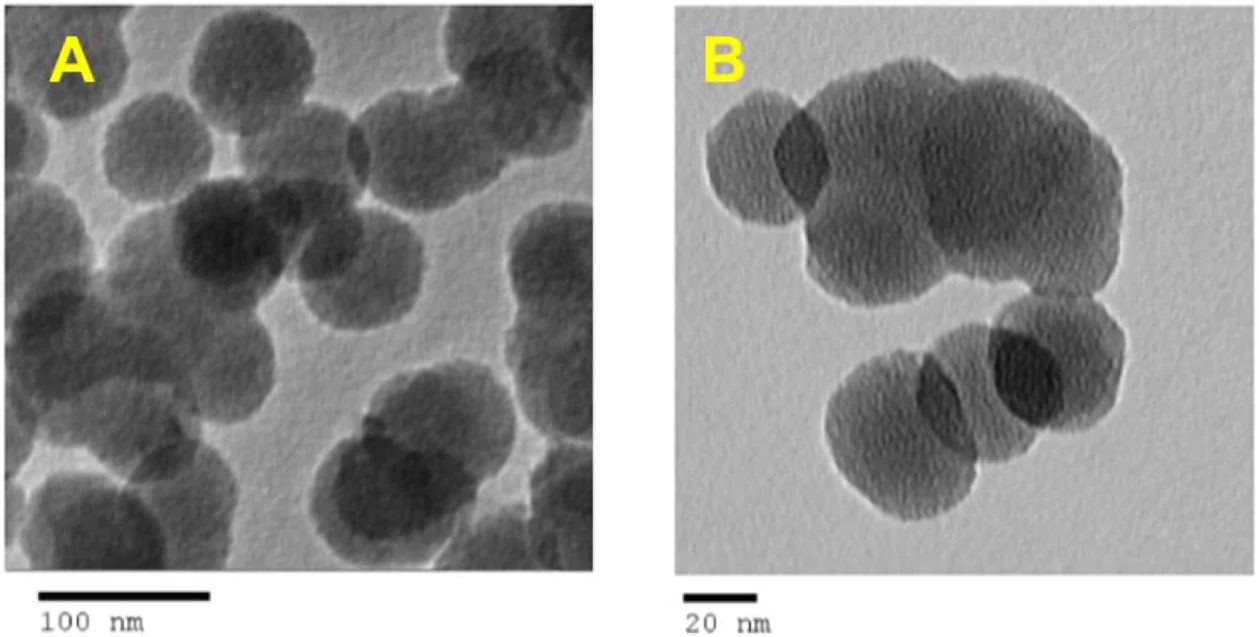
TEM images of MSNs (panel **A**) and Si-NQMP-ASA nanoparticles (panel **B**).

The efficiency of the photocleavage of the PLL **3e** was first tested in homogeneous solution. 0.5 mM solution of **3e**-ASA ester (**6**) in (1:1 H_2_O/CH_3_CN) was irradiated with 300 nm fluorescent lamps, while the formation of ASA and the regeneration of **3e** was monitored by HPLC (Scheme 9). The complete consumption of **6** and the quantitative formation of ASA were achieved after 8 min of exposure. The yield of **3e** was only ∼25% due to a subsequent photoreaction of the PLL. Photorelease of ASA was also quantitative under 350 nm light but required 15 min of exposure.

**Scheme 9:**
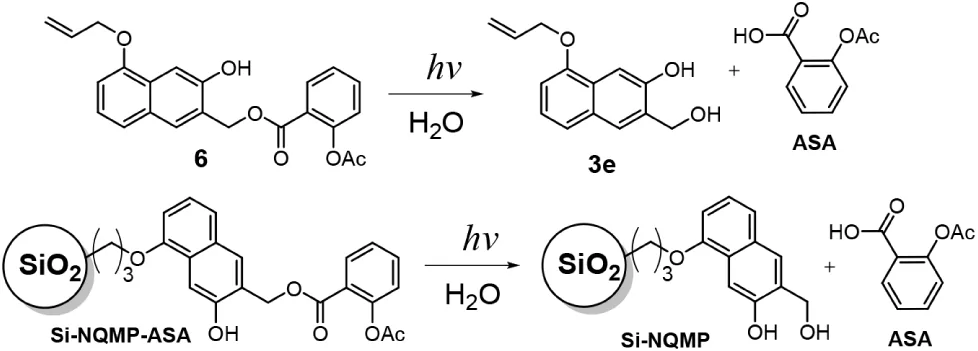
Photocleavage of **3e**-ASA Ester (**6**) and Photorelease of Acetylsalicylic Acid from MSNPs

For the investigation of the photorelease of acetylsalicylic acid from silica nanoparticles, a suspension of **Si-NQMP-ASA** in PBS was irradiated at 300 or 350 nm under constant stirring (Scheme 9) and the concentration of ASA in the supernatant was periodically measured by HPLC (Figure 6). The saturation of substrate release under 300 nm irradiation is achieved at ∼45 min, while under 350 nm light, even after 2.5 h of exposure, the concentration of ASA barely reaches 85% of the maximum release. As mentioned above, the slower photocleavage of the NQMP linker under 350 nm irradiation is due to the lower extinction coefficient of the NQMP chromophore at this wavelength (Figure S5).

**Figure 6:**
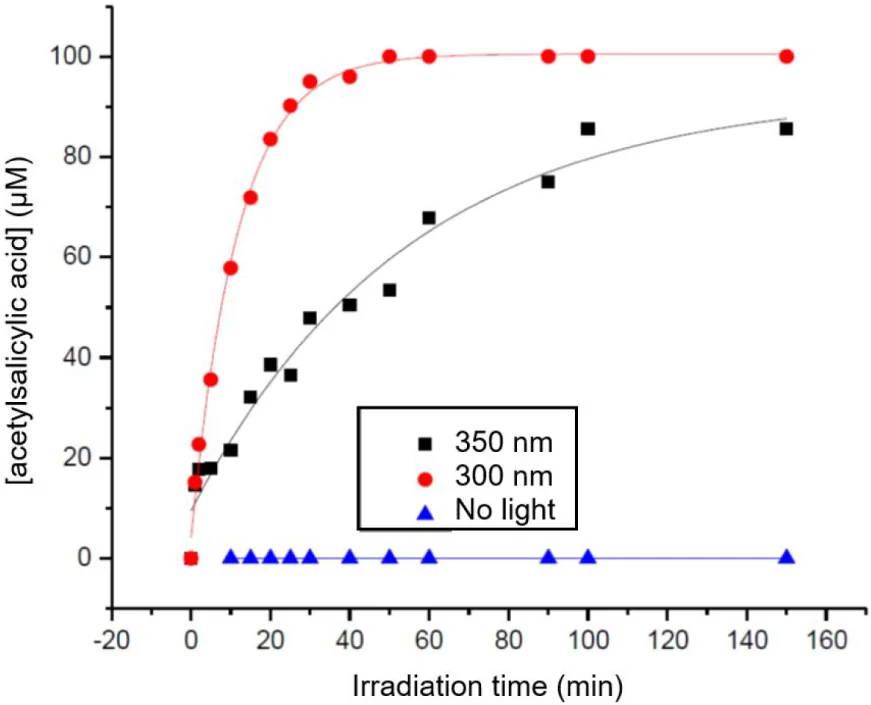
Photorelease of acetylsalicylic acid from a suspension of **Si-NQMP-ASA** in PBS; concentration of ASA in the supernatant solution under 300 nm (red circles) and 350 nm irradiation (black squares), or during incubation in the dark (blue triangles).

The photorelease of ASA from **Si-NQMP-ASA** requires a much longer irradiation time than does photocleavage of **6** in solution. We believe that the slower release of ASA from MSNPs is mostly due to the reversibility of the o-NQM formation. In aqueous solution, rapid hydration of the **o-NQMP** intermediate outcompetes the reverse reaction. Inside MSNP pores, local water concentration is lower, and recombination of o-NQM with ASA back to Si-NQMP-ASA reduces the quantum yield of ASA photorelease.

The concentration of aspirin at the release endpoint (under 300-nm irradiation) was 95 μM, as derived from the calibration curve. Therefore, the release capacity of **Si-NQMP-ASA** nanoparticles in PBS is 12 mg (67 μmol) of aspirin per 1 g of **Si-NQMP-ASA** MSNPs. In a control experiment, a PBS suspension of **Si-NQMP-ASA** nanoparticles was stirred in the dark for 2.5 h, but no acetylsalicylic acid was detected in the supernatant solution (Figure 6).

### Photorelease of *D*-Biotin from the Surface-Derivatized MSNPs

To create MSNPs with a derivatized surface but native silica pores, we explored a two-step derivatization. First, silica nanoparticles (SNPs) prepared by a sol–gel procedure were derivatized with amino or azide groups by treating the nanoparticles in dry DMF with 3-aminopropyltriethoxysilane and 3-azidopropyltriethoxysilane, respectively.^20^ NH_2_-SNPs and N_3_-SNPs were treated with hydrochloric acid to remove the surfactant and create pores. The mesoporous structure of NH_2_-MSNPs and N_3_-MSNPs can be seen in the TEM image (Figure S4). The presence of amino groups on the NH_2_-MSNPs surface was confirmed by the fluorescamine assay, while IR spectroscopy was employed to confirm azide functionalization of N_3_-MSNPs.^20^ At pH 7.4, the almost neutral zeta potential of unmodified MSNPs (−0.8 mV) becomes positive for NH_2_-MSNPs (+38.0 mV) and N_3_-MSNPs (+27.6 mV).^20^

The copper-catalyzed click reaction between N_3_-MSNPs and the propargyl-NQMP-Biotin photolabile linker (**3d**) produced **Si-NQMP-Biotin 1** MSNPs (Scheme 10). The TEM image confirms the preservation of mesoporous morphology in **Si-NQMP-Biotin 1** (Figure 7).^20^ Two catalytic systems were tested for the CuAAC biotinylation of N_3_-MSNPs: CuBr­(PPh_3_)_3_/Et_3_N in THF and CuSO_4_/sodium ascorbate in DMF. An alternative synthesis of biotinylated MSNPs was achieved by EDC coupling of PLL **3c** with NH_2_-MSNPs to yield **Si-NQMP-Biotin 2** (Scheme 10). The Cu­(I)-catalyzed reaction produced the highest degree of derivatization (*vide infra*).^20^

**Figure 7:**
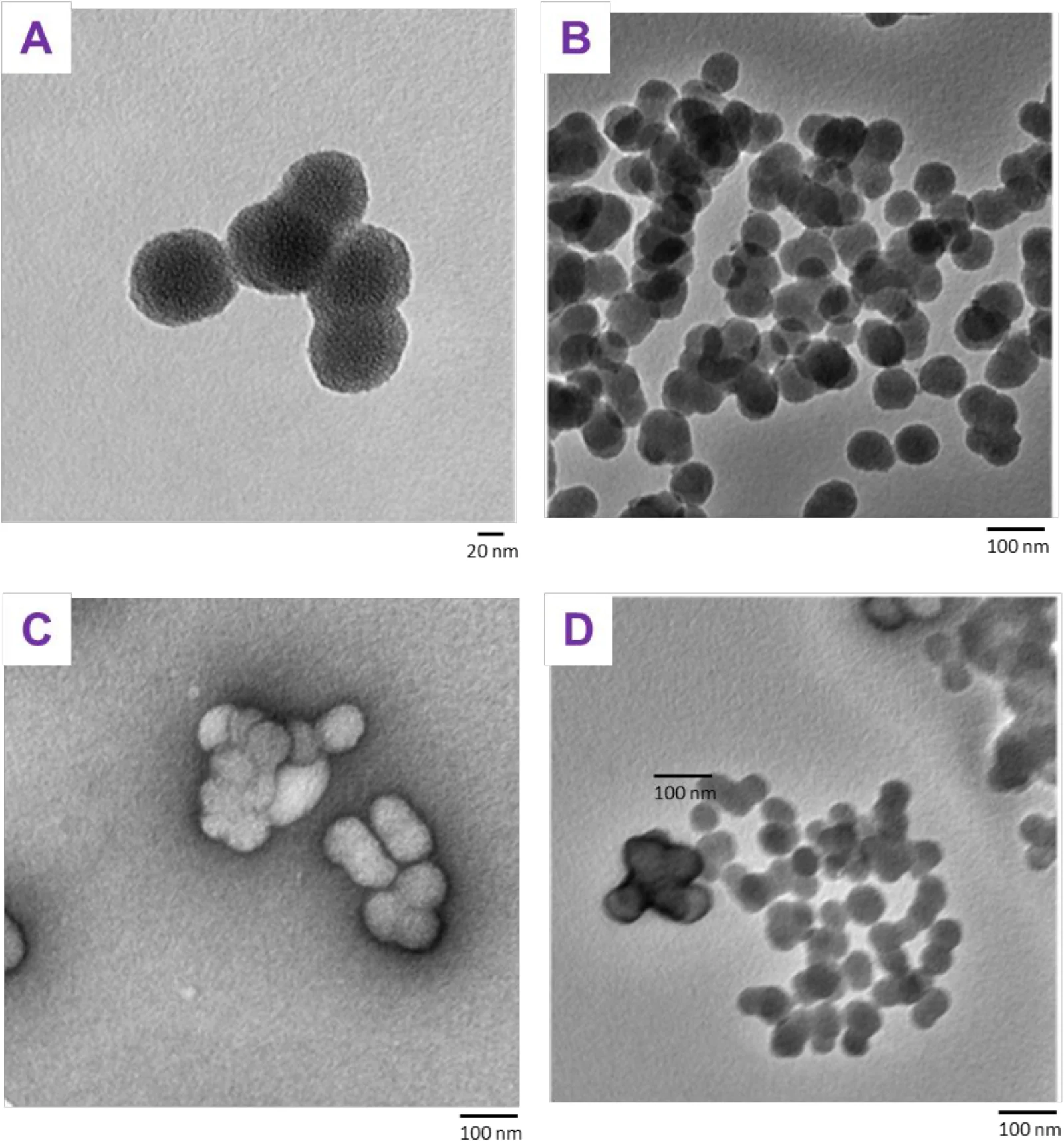
TEM images of biotinylated MSNPs treated with uranyl acetate negative stain. Panels A and B show **Si-NQMP-Biotin** MSNPs; panel C shows **Si-NQMP-Avidin** MSNPs; panel D shows **Si-NQMP-Avidin** MSNPs after 30 min of irradiation with 350 nm light.

**Scheme 10:**
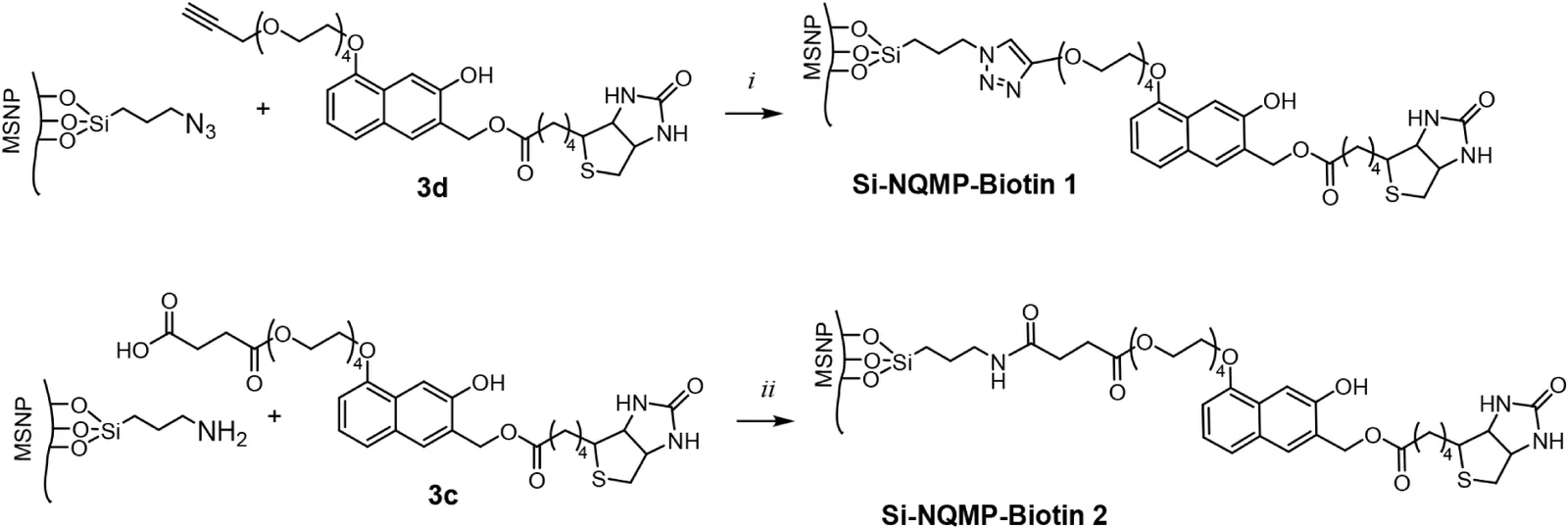
Biotinylation of Mesoporous Silica Nanoparticles (MSNPs) with Propargyl-NQMP-Biotin (**3d**) and Carboxylate-NQMP-Biotin (**3c**) Photolabile Linkers^a^

a Reagents and conditions: (i) CuBr­(PPh_3_)_3_, THF, TEA; or CuSO4, sodium ascorbate, DMF; (ii) EDC, DMAP, DMF.

The photorelease of biotin from **Si-NQMP-Biotin 1** and **Si-NQMP-Biotin 2** MSNPs was achieved by irradiation of the nanoparticle suspension in PBS with 350 nm lamps. Photohydrolysis of the NQMP photolinker (Scheme 2) produced **Si-NQMP 1** and **2** and *D*-biotin (Scheme 11).

**Scheme 11:**
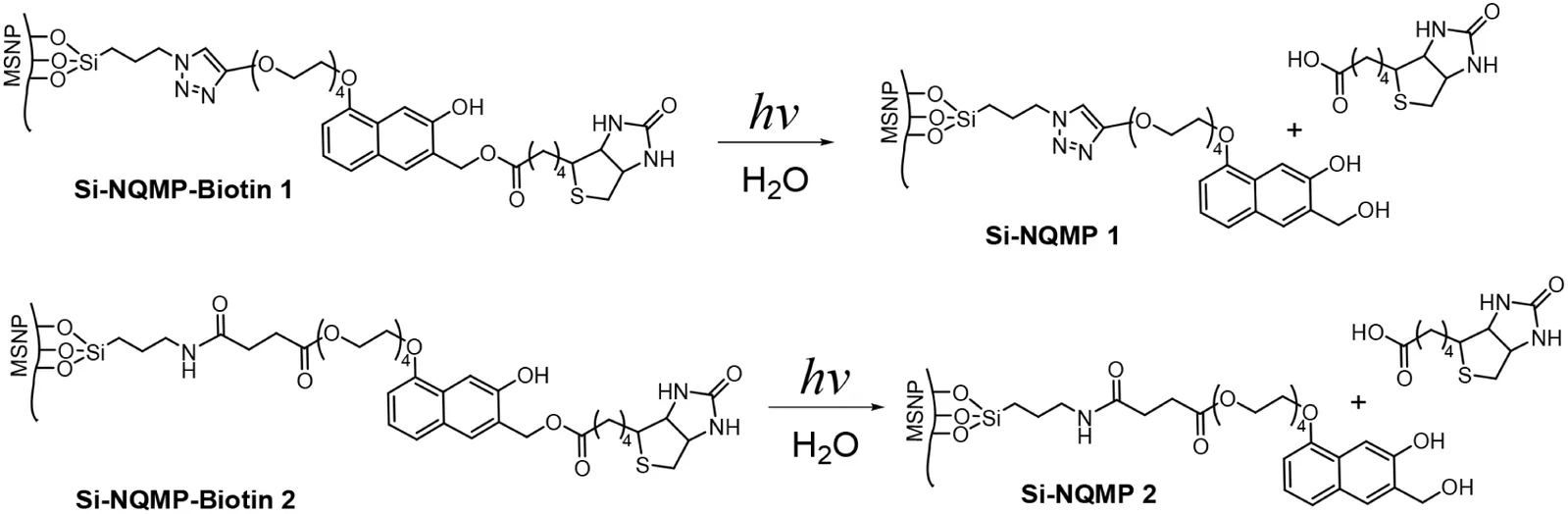
Photo-Release of Biotin from **Si-NQMP-Biotin 1** and **2** MSNPs.

The amount of biotin released from **Si-NQMP-Biotin 1** and **2** was determined using the HABA/Avidin assay.^20^ Specifically, suspensions of biotinylated MSNPs were irradiated with 350 nm light for 1 h; MSNPs were separated by centrifugation, washed with DMF, and the concentration of biotin in the combined supernatant and wash solutions was measured by the decrease in absorbance of the HABA/Avidin complex at 500 nm.^20^ The highest yield of photoreleased biotin, ∼0.92 μmol (0.23 mg) of biotin per 1 g of MSNPs, was observed in the case of **Si-NQMP-Biotin 1** MSNPs produced by CuBr­(PPh_3_)_3_-catalyzed click reaction. These MSNPs were employed in subsequent studies. Irradiation of **Si-NQMP-Biotin 1** MSNPs for 5 min released ∼0.47 μmol, and for 15 min, 0.81 μmol of biotin per 1 g **of Si-NQMP-Biotin 1** MSNPs.^20^ Thus, 50% of the biotin was released in the first 5 min of irradiation, and after 15 min of irradiation, the yield of the photorelease reached 90%. No biotin was detected in the supernatant solution of **Si-NQMP-Biotin 1** MSNPs that were irradiated for 1 h, washed, and irradiated for a second time. This observation suggests that all biotin was cleaved from the MSNPs during 60 min of exposure to 350 nm light. Overnight stirring of a PBS suspension of **Si-NQMP-Biotin 1** MSNPs in the dark produced no detectable amount of biotin in the supernatant solution. It is important to note the 70-fold loading difference between surface-derivatized (0.92 μmol g^–1^, **Si-NQMP-Biotin**) and pore-derivatized (67 μmol g^–1^, **Si-NQMP-ASA**, *vide supra*) MSNPs.

### Photorelease of Avidin from the Biotin-Derivatized MSNPs

One of the strategies for targeted drug delivery using MSNPs is to load the particles with a bioactive compound and cap the pores. The caps are removed in the target tissue, and the drug is released.^24^ Previously, the utility of three photochemical reactions for light-controlled MSNP drug delivery was explored: azobenzene photoisomerization,^25^ reversible coumarin dimerization,^4^ ^c^ and photohydrolysis of coumarin-4-methyl PLL.^4^ ^d^ We decided to test the utility of NQMP-based PLLs for this purpose using a large protein to block the surface of nanoparticles. The very high affinity of avidin for biotin allowed us to coat biotinylated nanoparticles with this protein. Thus, the **Si-NQMP-Biotin** MSNPs were treated with avidin, resulting in the formation of a surface layer of protein on MSNPs (**Si-NQMP-Avidin** Scheme 12).^20^ Exposure of an aqueous suspension to UVA light resulted in the cleavage of the NQMP-biotin ester and the release of avidin from the surface of MSNPs (Scheme 12).

**Scheme 12:**
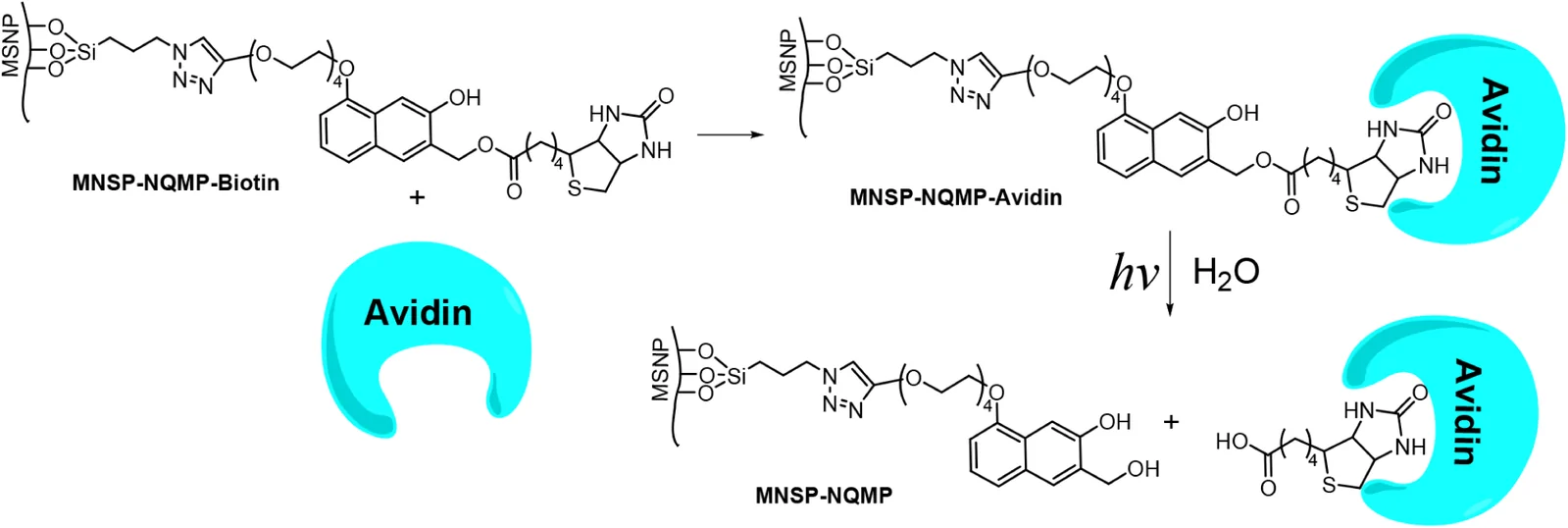
Immobilization and Photo-Release of Avidin on **Si-NQMP-Biotin** MSNPs

The avidin coating of MSNPs and the photorelease of the protein were monitored by TEM using a uranyl acetate negative stain (Figure 7). **Si-NQMP-Biotin** MSNPs and **Si-NQMP-Avidin** MSNPs, before and after irradiation, were deposited on the TEM grids and treated with a 2% solution of uranyl acetate in PBS. The **Si-NQMP-Biotin** MSNPs appear darker than the background in TEM images, as the stain penetrates the pores of the nanoparticles (Figure 7A,B). Avidin-coated MSNPs (**Si-NQMP-Avidin**) are brighter than the background, as the protein layer prevents the stain from penetrating the MSNPs, but the stain is still present on the surface of the grid (Figure 7C). Upon irradiation, the avidin layer is removed from the particles, and the MSNPs are again darker than the background (Figure 7D).

### Photorelease of Doxorubicin from MSNPs

To explore the feasibility of the light-directed release of drugs from NQMP-avidin-capped MSNPs, the **Si-NQMP-Biotin** MSNPs were loaded with doxorubicin (DOX) and coated with an avidin layer.^20^ The release of DOX from the resulting drug-loaded particles kept in the dark and under 350 nm irradiation was monitored by UV–vis and fluorescence spectroscopy. In a control experiment, an 86 μM solution of DOX in PBS was irradiated for 30 min with 350 nm light at r.t. No significant changes were observed in the UV spectra before and after irradiation, suggesting stability of the drug under these conditions. **Si-NQMP-Biotin 1** MSNPs were loaded with DOX by incubating the nanoparticles in a buffered aqueous solution of the drug. The amount of DOX absorbed by MSNPs was determined by measuring the reduction in the concentration of DOX in solution by following the absorbance band at 480 nm. In neutral solutions (pH = 7.41), the loading capacity of **Si-NQMP-Biotin 1** MSNPs was found to be 312 mg (0.57 mmol) DOX per g of MSNPs, while in basic solutions (pH = 9.28) it increased to 726 mg (1.3 mmol) per g of nanoparticles.

Avidin solution was added to the suspension of DOX-loaded **Si-NQMP-Biotin 1** MSNPs in PBS; the nanoparticles were incubated for 4 h, separated by centrifugation, and washed with PBS. The suspension of MSNPs was placed in a dialysis tube, and the release of DOX was monitored by the emission intensity at 595 nm (excitation at 480 nm) of the outer solution of the dialysis kit (Figure 8). Doxorubicin was released slowly from DOX-loaded **Si-NQMP-Avidin** MSNPs in the dark (blue circles in Figure 8). After 4 h of dialysis, half of the samples were irradiated for 15 min with 350 nm light and returned to dialysis (red circles). As seen in Figure 8, the rate of release and the overall amount of doxorubicin released from MSNPs at the saturation point almost doubled.

**Figure 8:**
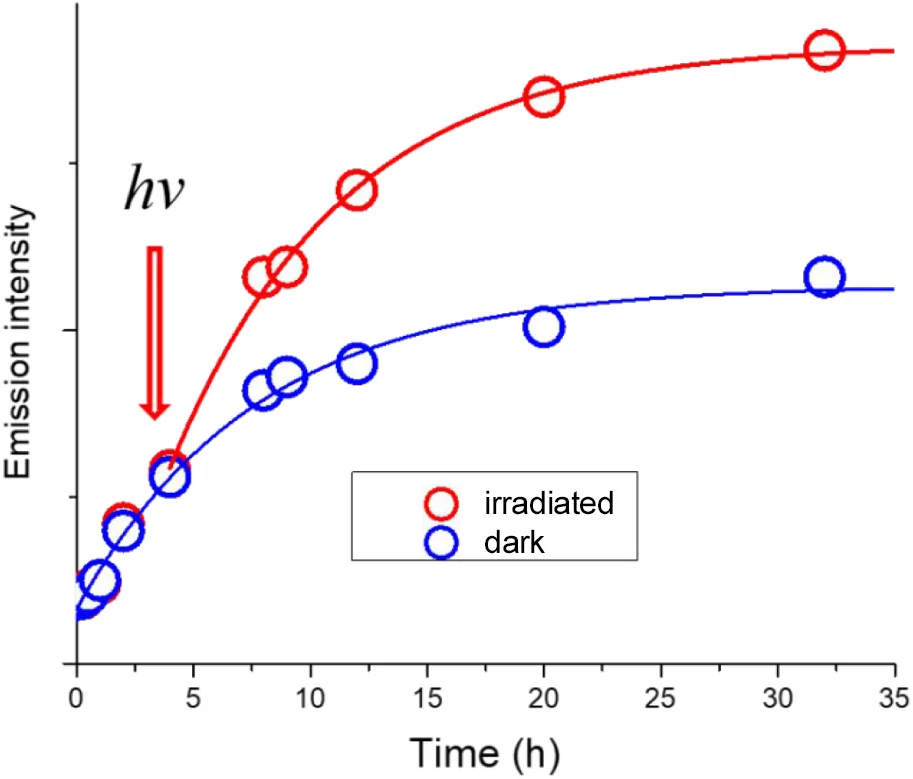
Release of doxorubicin (DOX) from the drug-loaded **Si-NQMP-Avidin** MSNPs, monitored by emission at 595 nm (excitation at 480 nm). Blue circles represent the emission of DOX from MSNPs incubated in the dark, while red circles represent the emission of DOX from MSNPs irradiated for 15 min at 350 nm after 4 h of dialysis.

This experiment demonstrates the potential utility of using a photoremovable protein layer to reduce the rate of release of the payload compound from the pores of MSNPs, but it is far from an ideal on–off system. We are currently working on the optimization of the MSNPs’ biotin-derivatization density to achieve higher drug-retention parameters for **Si-NQMP-Avidin** MSNPs.

### Syntheses of Photolabile Linkers

Synthesis of oNQMP-based PLLs **3a**–**e** starts with selective Williamson etherification of the 5-hydroxy group in methyl 3,5-dihydroxy-2-naphthoate (**7**) with an appropriate linker, i.e., propargyl-EG_4_ for **3f** and **3d**, trityl-EG_4_ for **3c**, and O-allyl for **3e** (**8**, Scheme 13).^20^ LAH reduction of the ester group in **8** produces oNQMP derivatives **9a**–**c** and PLL **3e**. For the preparation of **3a** and **3b**, the TBDMS protection of the ethylene glycol terminus in **9a** is removed by hydrofluoric acid in aqueous acetonitrile, and the o-hydroxybenzyl alcohol functionality in the resulting **10** is converted into a dimethyl ketal by the acid-catalyzed reaction with 2,2-dimethoxypropane to give **11a**. The azide group is installed using a previously reported procedure, yielding the precursor **11c** (Scheme 13).^20,26^

**Scheme 13:**
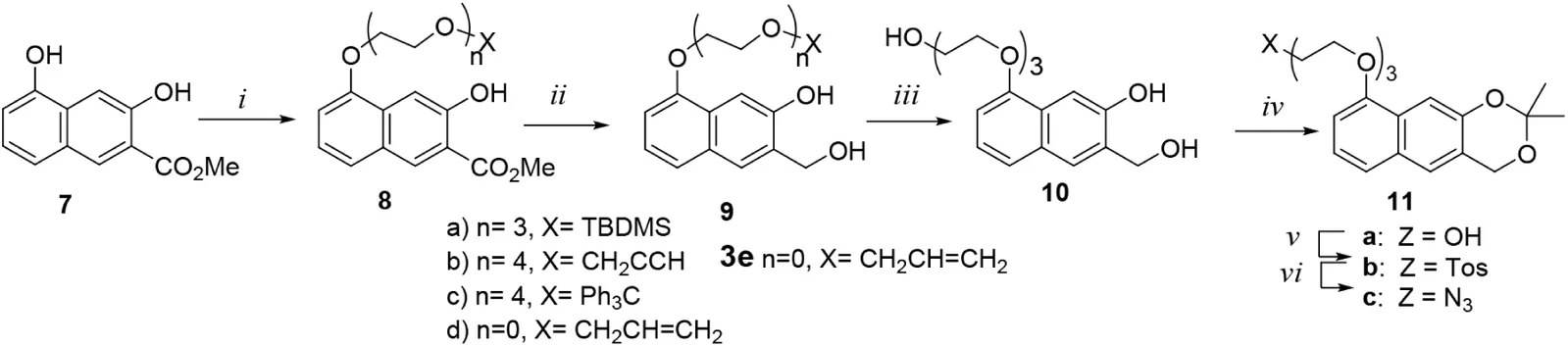
Synthesis of NQMP-PLL Precursors and PLL **3e** ^a^

a Reagents and conditions: (i): (a) TBDMS-EG3-I, (b) Propargyl-EG4-I, (c) Trityl-EG4-I, (d) Br-CH_2_CH=CH_2_; K_2_CO_3_, acetone, reflux, 50–80%; (ii) LiAlH_4_, THF, 86–98%; (iii) HF/CH_3_CN (aq., quant.); (iv) TsOH, 2,2-dimethoxypropane, acetone, 90%; (v) TsCl, pyridine, DCM, 82%; (vi) NaN_3_, DMF, 60 °C, 80%.

For the preparation of PLL **3a**, the ketal protection in **11c** is removed by acid-catalyzed solvolysis in MeOH with Amberlyst-15 to give N_3_-EG_3_-NQMP (**12**). Both phenolic and benzylic hydroxy groups are converted into TBDMS ethers (**13a**). The benzylic position is then selectively deprotected with CeCl_3_, and the resulting alcohol **13b** is coupled with propargyl bromide under Williamson conditions to give propargyl ether **13c**. LAH treatment of the latter allows for simultaneous reduction of the azide functionality and removal of the TBDMS protection, resulting in the formation of the target NH_2_-EG_3_-NQMP-propargyl photolabile linker **3a** (Scheme 14).^20^

**Scheme 14:**
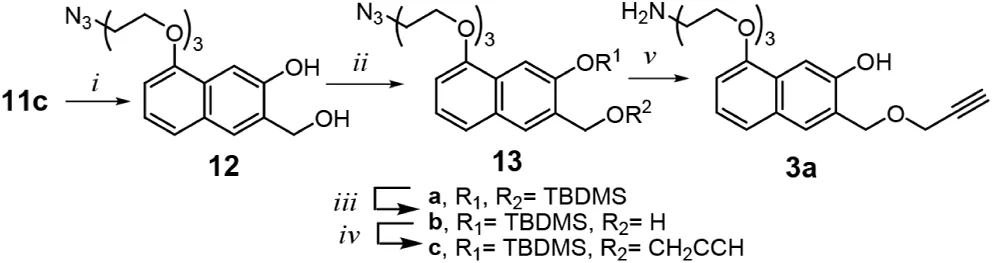
Synthesis of NH_2_-EG_3_-NQMP-Propargyl Photolabile Linker (**3a**)­^a^

a Reagents and conditions: (i) Amberlyst-15, MeOH; (ii) TBDMSCl, imidazole, DMAP, DMF, 80% over two steps; (iii) CeCl_3_, CH_3_CN, 85%; (iv) NaH, propargyl bromide, THF, 81%; (v) LiAlH_4_, THF, 94%.

Ketal **11c** also served as a precursor to ADIBO-NQMP–OH PLL (**3b**). The azido moiety in **11c** was reduced to an amino group to give **14**, which was, in turn, coupled with ADIBO acid **15** under EDC/DMAP conditions to give **16**. Mild hydrolytic removal of the acetal protecting group yielded the target ADIBO-NQMP-OH photolabile linker in excellent yield (**3b**, Scheme 15).^20^

**Scheme 15:**
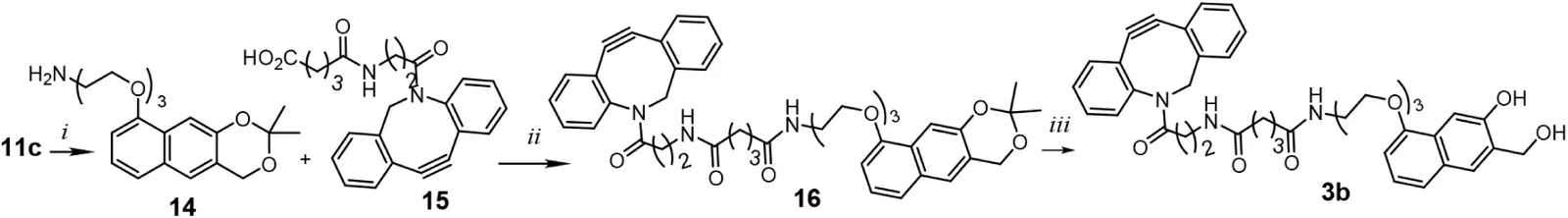
Synthesis ADIBO-NQMP Photolabile Linker (**3b**)­^a^

a Reagents and conditions: (i) LAH, THF; (ii) EDC, DMAP, THF, 85% over two steps; (iii) Amberlyst-15, MeOH, 93%.

The biotinylated linker **3c** was prepared via two consecutive esterification reactions. First, the benzylic alcohol moiety of trityl-protected EG_4_-NQMP (**9c**) was EDC-coupled to *D-*biotin yielding **17a**. The TFA-catalyzed removal of trityl protection, followed by reaction of the resulting **17b** with succinic anhydride, produced carboxyl-EG_4_-NQMP-biotin PLL (**3c**) in good yield (Scheme 16).^20^

**Scheme 16:**
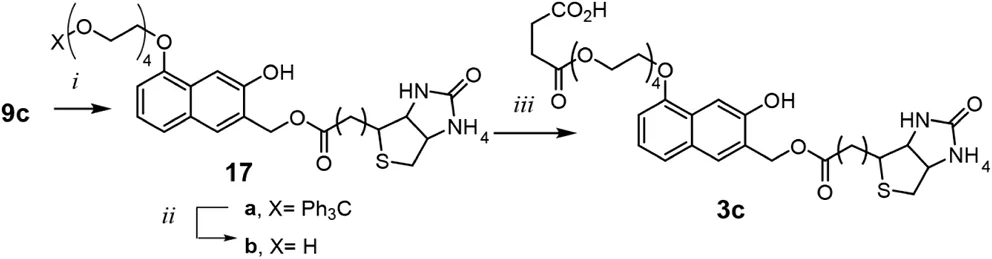
Synthesis of Carboxyl-EG_4_-NQMP-Biotin (**3c**)­^a^

a Reagents and conditions: (i) D-biotin, EDC, DMAP, DMF, 71%; (ii) TFA, DCM, 94%; (iii) succinic anhydride, TEA, DCM, 80%.

Finally, propargyl-EG_4_-NQMP-ADIBO (**3f**) and propargyl-EG_4_-NQMP-Biotin (**3d**) were synthesized starting from propargyl-EG_4_-NQMP-OH (**9b**) by EDC/DMAP coupling with ADIBO acid (**15**) and *D*-biotin, respectively (Scheme 17).^20^

**Scheme 17:**
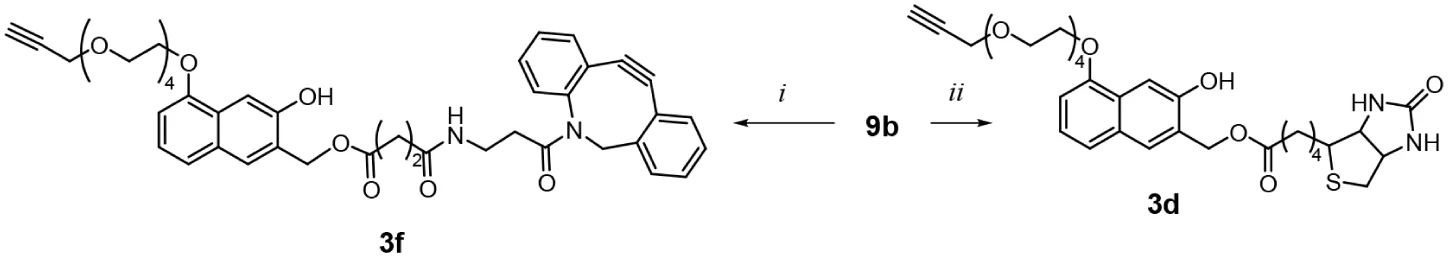
Synthesis of Propargyl-NQMP-ADIBO (**3f**) and Propargyl-NQMP-Biotin (**3d**) Photolabile Linkers^a^

a Reagents and conditions: (i) 15, EDC, DMAP, DMF, 46%; (ii) D-biotin, EDC, DMAP, DMF, 40%.

## Conclusions

We have developed a versatile family of heterobifunctional photolabile linkers based on the (3-hydroxy-5-alkoxynaphthalen-2-yl)­methyl (NQMP) chromophore for the efficient immobilization and light-triggered release of substrates from solid supports. The photocleavage of the NQMP photolabile linkers is characterized by high quantum and chemical yields of the process, which are insensitive to the basicity of the leaving group, as well as by fast photorelease. The modular linker design combines rapid and high-yielding NQMP photochemistry with orthogonal conjugation chemistries, including CuAAC and SPAAC click reactions, amide coupling, and biotin–avidin binding, enabling straightforward attachment of a broad range of payloads and surfaces. The synthetic strategy presented in this report provides convenient access to linkers bearing complementary reactive handles that can be adapted to diverse immobilization and bioconjugation applications.

The utility of these linkers was demonstrated by the efficient immobilization and high-to-quantitative photorelease of fluorescent dyes, acetylsalicylic acid, biotin, avidin, and doxorubicin from silica microbeads and mesoporous silica nanoparticles. Pore-functionalized mesoporous nanoparticles enable high substrate loading. The combination of efficient synthesis, orthogonal conjugation chemistry, and robust photochemical performance establishes NQMP-based linkers as practical and broadly applicable platforms for the development of photoresponsive materials, bioconjugates, and drug delivery systems.

Future studies will focus on extending the NQMP platform to longer-wavelength-responsive photolabile linkers and evaluating their performance in biologically relevant environments, where increased tissue penetration and reduced phototoxicity will further expand their utility.

## Supplementary Material



## Notes

Experimental procedures, synthesis of and characterization of compounds, relevant UV and IR spectra, and TEM images (PDF)
