# Supplementary material for: Clickable Photolabile Linkers for Convenient Immobilization and Light-Controlled Release of Substrates from Solid Support
Source: J Am Chem Soc. 2026 Oct 14;148(42):46397–409. doi: 10.1021/jacs.6c18307 (PMC13721149; doi:10.1021/jacs.6c18307)
Supplement: Supplementary file 1 [file ja6c18307_si_001.pdf]

Supporting Information

Clickable Photolabile Linkers for Convenient Immobilization and Light-controlled Release of Substrates from Solid Support

Selvanathan Arumugam, Nannan Lin, Chen Zhao, Jin Xie, and Vladimir V. Popik*

Department of Chemistry, University of Georgia, GA 30602, USA

vpopik@uga.edu

Table of contents:

Experimental……………..……………………..……………………...……………………………..S2

Preparation of the photolabile linkers ………………………………………………………S9

Experimental

General Methods. Flash chromatography was performed using 40-63 μm silica gel. All NMR spectra were recorded in $CDCl_3$ using 400 MHz instrument and chemical shifts were referenced to TMS unless otherwise noted. Solutions for photochemical reactions were prepared using HPLC grade water and acetonitrile. Electronic spectra and scanning kinetics were recorded using Cary 60 UV-Vis spectrometer.

Photolytic experiments. Photo-release experiments were performed by irradiating rapidly stirred suspensions of derivatized silica microbeads or mesoporous silica nanoparticles (1 – 4 mg mL^{-1}) in a quartz vessel using a Luzchem LZC-4 photochemical reactor equipped with 8 (18 W) fluorescent UV lamps (300 or 350 nm).[1] The approximate irradiance of the 300 nm setup is 6.9 mW cm^{-1} and of the 350 nm setup is 6.7 mW cm^{-1}.[1] The quantification of payload release, as well as calibration plot points, were all measured in triplicate.

Materials: DCM, DMF, and THF were purified by passing through the alumina column before use. HPLC-grade hexanes, ethyl acetate, and methanol were dried over anhydrous sodium sulfate or 4Å molecular sieves. Karstedt's catalyst was obtained from Gelest Inc., all other reagents were purchased from Sigma or Fisher, unless noticed otherwise.

Carboxy-functionalized silica microbeads (average size 1 μm) were purchased from Polysciences Inc. The degree of carboxylate functionalization of silica microbeads was determined by potentiometric titration. A stirred aqueous suspension of silica microbeads in H-form was titrated with a 0.0020 N solution of NaOH, and pH was monitored by a pH meter. The fit of experimental data on pH dependence on titrant volume to a sigmoidal function allows for the determination of equivalency point at 95 μL. This value corresponds to ~0.01 μmol of carboxylate groups per mg of silica beads.

Immobilization of Texas Red dye on Silica Microbeads

Preparation of **Si-NQMP-TR** (Scheme 4). EDC (66 mg, 0.34 mmol) and a catalytic amount of DMAP were added to a dispersion of carboxyl functionalized silica microspheres (2 g) in 20 mL of anhydrous DMF, followed by the addition of a solution of PLL **3a** (72 mg, 0.2 mmol) in 0.5 mL of DMF. The mixture was stirred for 48 h at r.t., and the particles were isolated by centrifugation (5,000 rpm, 10 min). The resulting silica beads (**Si-3a**) were washed with DMF trice and dried under a flow of nitrogen.

A solution of Texas Red azide (7 mg, 0.01 mmol) was added to a stirred suspension of **Si-3a** microbeads (200 mg) in 20 mL of anhydrous DMF. A stock solution of a catalyst containing 0.5

mL of DMSO, PMDETA (423 μL, 2.03 mmol), CuBr (39 mg, 0.28 mmol), and $CuCl_2$ (7.26 mg, 0.05 mmol) in 10 mL DMF was separately prepared. About 100 μL of the catalytic solution was added to the dispersion containing the dye and **Si-3a**. The mixture was stirred for 2 h at r.t. and the particles were isolated by centrifugation (5,000 rpm, 10 min). The resulting silica beads (**Si-NQMP-TR**) were washed with DMF until there was no dye detected in the wash solvent by UV spectroscopy and dried under a flow of nitrogen. The fluorescent dye - derivatized silica beads (**Si-NQMP-TR**) were spin-coated on a glass slide from an acetonitrile suspension using two concentrations (2 g L^{-1} and 0.02 g L^{-1}) and the images were recorded via Olympus IX71 inverted fluorescence microscope using excitation at 567 nm. Lumps of silica particles exhibit bright fluorescence (Figure S1).

(a) (b)

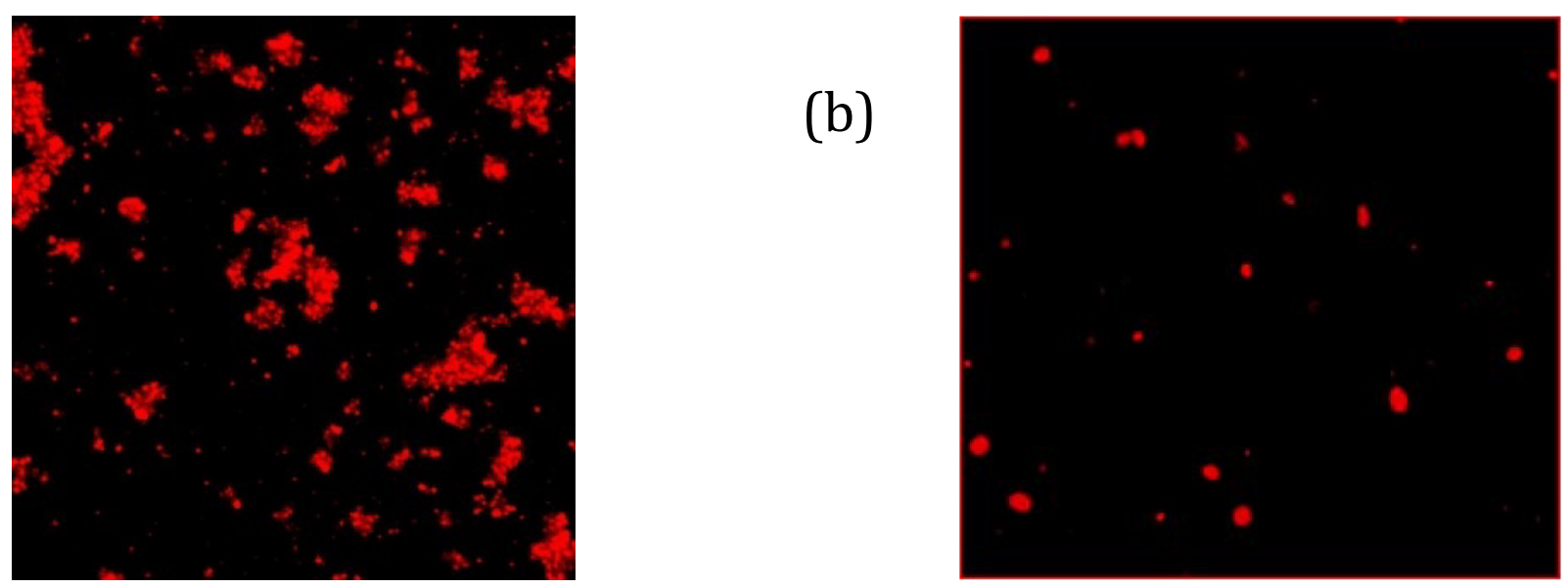

Figure S1. Fluorescent microscope images of **Si-NQMP-TR** silica microbeads spin coated on a glass slide (a) from 2 mg/mL and (b) from 0.02 mg/mL suspension

Si-TR microbeads were by the same sequence of reactions only using propargyl amine instead of PLL **3a** (Scheme 4).

Immobilization of Fluorescein on Silica Microbeads

Preparation of **Si-NQMP-Fluor** (Scheme 6). A solution of *N*-hydroxy succinimide (11.5 mg, 0.1 mmol), EDC (33 mg, 0.1 mmol) and a catalytic amount of DMAP in 1 mL of anhydrous DMF was added to a stirred suspension of carboxyl functionalized silica microbeads (1g) in 20 mL of anhydrous DMF. The mixture was stirred for 48 h at r.t. and the particles were isolated by centrifugation (5,000 rpm, 10 min). The resulting silica beads (**Si-NHS**) were washed with DMF three times and dried under a flow of nitrogen.

A solution of PLL **3b** (70 mg, 0.1 mmol) and triethylamine (10μL) in 2 mL of anhydrous THF was added to the stirred suspension of **Si-NHS** (1 g) in 10 mL anhydrous THF and stirred for 48 h at r.t. The resulting silica beads (**Si-NQMP-ADIBO**) were isolated by centrifugation (5,000 rpm, 10 min), washed with 1% HCl, DMF, and dried under a flow of nitrogen.

A solution of 6-carboxyfluorescein azide (5 mg, 0.011 mmol) was added to a stirred suspension of **Si-NQMP-ADIBO** (200 mg) in 20 mL of anhydrous DMF. The mixture was stirred for 2 h at r.t. and the particles were isolated by centrifugation (5,000 rpm, 10 min). The **Si-NQMP-Fluor** beads were washed with DMF till there was no dye in the wash solvent (by UV spectroscopy) and dried under a flow of nitrogen.

Preparation of **Si-Fluor** (Scheme 6). A solution of ADIBO-amine (**5**, 27 mg, 0.1 mmol) and triethylamine (10μL) in 2 mL of anhydrous THF was added to the stirred suspension of **Si-NHS** (1 g) in 10 mL anhydrous THF and stirred for 48 h at r.t. The resulting silica beads (**Si-ADIBO**) were isolated by centrifugation (5,000 rpm, 10 min), washed with 1% HCl, DMF, and dried under a flow of nitrogen.

A solution of 6-carboxyfluorescein azide (5 mg, 0.011 mmol) was added to a stirred suspension of **Si-ADIBO** (200 mg) in 20 mL of anhydrous DMF. The mixture was stirred for 2 h at r.t. and the particles were isolated by centrifugation (5,000 rpm, 10 min). The **Si-Fluor** beads were washed with DMF till there was no dye in the wash solvent (by UV spectroscopy) and dried under a flow of nitrogen.

Preparation of Mesoporous Silica Nanoparticles (MSNPs).[2] Tetraethyl orthosilicate (TEOS, 3.34 g, 16.1 mmol) was slowly added to a vigorously vortexed solution of cetyltrimethylammonium bromide (CTAB (2.19 g, 6.00 mmol) in 200 mL of 0.1 M biphosphate buffer (pH = 7.0) at 95°C. The reaction mixture was stirred for 8 h at 95°C and allowed to cool down to r.t. The resulting silica particles were separated and collected by centrifugation (8000 rpm, 10 min) and washed twice with water and ethanol. The particles were re-suspended in 100 mL of 2% HCl in ethanol and refluxed under stirring for 12 h. The MSNPs were separated by centrifugation (8000 rpm, 10 min), washed twice with ethanol, and dried under reduced pressure. 700 mg MSNPs were isolated. The hydrodynamic diameter of the particles was 160±60 nm (by DLS) with almost neutral ζ-potential (-0.8 mV at pH = 7.4). The TEM imaging of MSNPs confirms their mesoporous structure with pore size of 2 – 3 nm and a core size of 30 to 80 nm (Figure 5).

Preparation of Acetyl Salicylic Acid – derivatized MSNPs (**Si-NQMP-ASA**, Scheme 8). Triethoxysilane (180 µl, 0.9 mmol) and 30 µl Karstedt's catalyst were added to a solution of **3e**-ASA ester (**6**, 160 mg, 0.43 mmol) in 10mL THF. The reaction mixture was stirred for 12 h at 60°C and stirred 12 hours. Solids were removed by filtration, solvent removed in vacuum, and the residue was quickly passed through a silica gel column (EtOAc:Hexanes 1:3) to yield 182 mg (80%) of triethoxysilyl-derivatized **6-TEOS** as a clear oil, which was immediately used in the next step.

A mixture of MSNPs (100 mg) and **6-TEOS** (100 mg) in 100 mL of toluene was sonicated for 20 min and then refluxed for 24 h with stirring. The resulting suspension was cooled down to r.t., **Si-NQMP-ASA** MSNPs were collected by centrifugation (8000 rpm, 10 min), washed twice with water and methanol, and dried under reduced pressure.

Preparation of Surface-derivatized MSNPs. Silica nanoparticles were prepared from TEOS in the presence of the surfactant as discussed above. After isolation and washing, 5 mL of 3-(aminopropyl)triethoxysilane (APTES, 21 mmol) were added to a stirred suspension of crude silica nanoparticles (400 mg) in 60 mL of anhydrous toluene. The reaction mixture was refluxed for 10 h, cooled down to r.t., and nanoparticles were collected by centrifugation (8000 rpm, 15 min). The particles were washed twice with EtOH, re-suspended in 200 mL of 2% HCl in ethanol and refluxed for 12 hours. The resulting NH_2-MSNPs were separated by centrifugation, washed twice with ethanol, and dried overnight under vacuum (0.2 mm Hg) at r.t.

The presence of amino groups on the surface of MSNPs was analyzed by the fluorescamine test. 50 µL of fluorescamine solution (7 mM in DMSO) were added to 150 µL of MSNPs suspension in PBS (1 g L^{-1}) and stirred for 30 s. Suspension of NH_2-MSNPs produced bright emission at 470 nm (under 365 nm excitation, Figure S2). No fluorescence was observed from the suspension of underivatized MSNPs, as well as from biotinylated MNSPs (**Si-NQMP-Biotin 2**, *vide infra*).

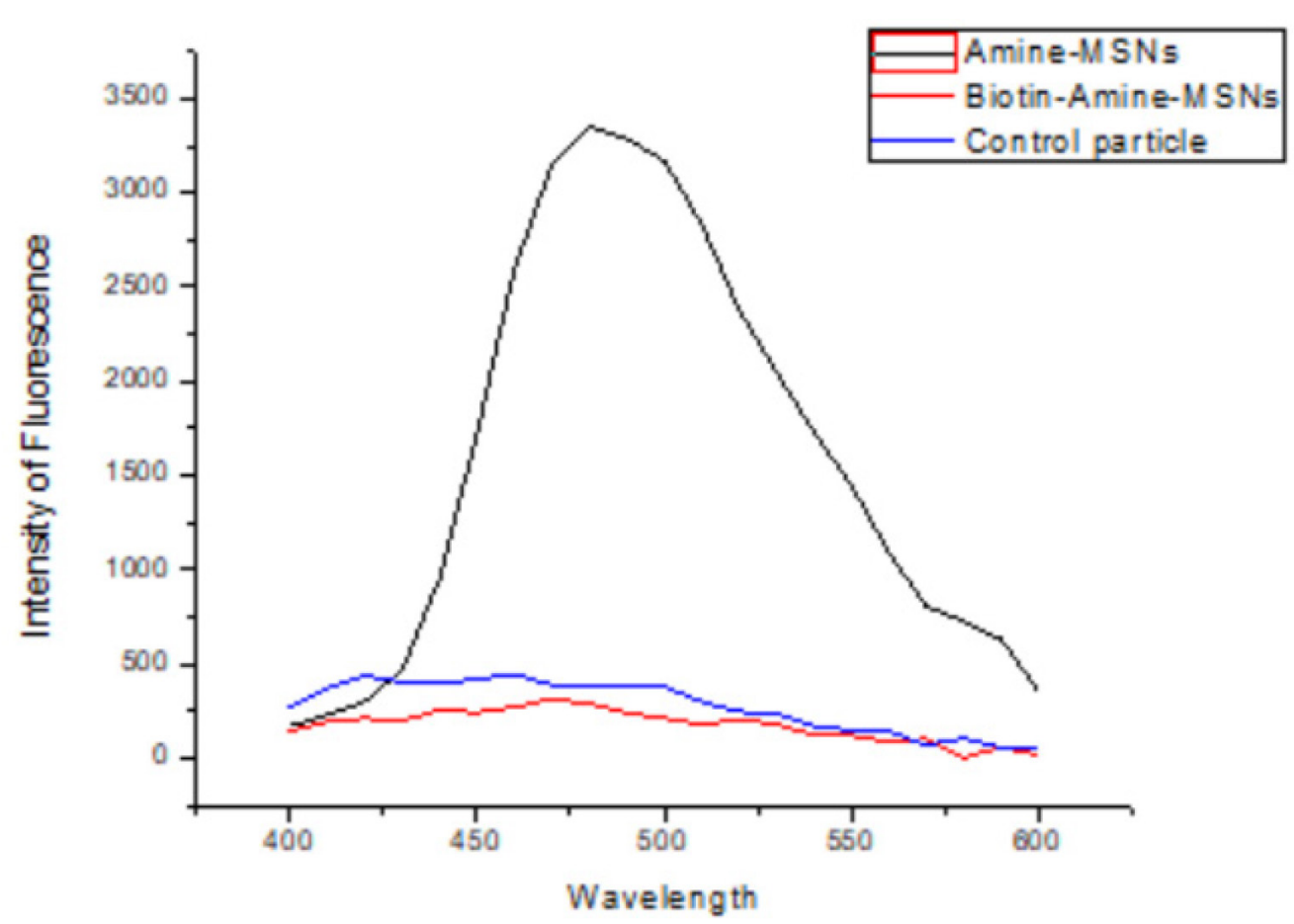

Figure S2. Fluorescamine test of NH_2-MSNPs (black line), underivatized MSNPs (blue) and **Si-NQMP-Biotin 2** (red line).

For the preparation of azide-derivatized MSNPs (**N_3-MSNPs**), 5 mL of 3-(azidopropyl)triethoxysilane instead of APTES in the derivatization procedure. The progress of the MNSP derivatization with an azide group and subsequent CuAAC-click biotinylation was monitored by IR spectroscopy (Figure S3).

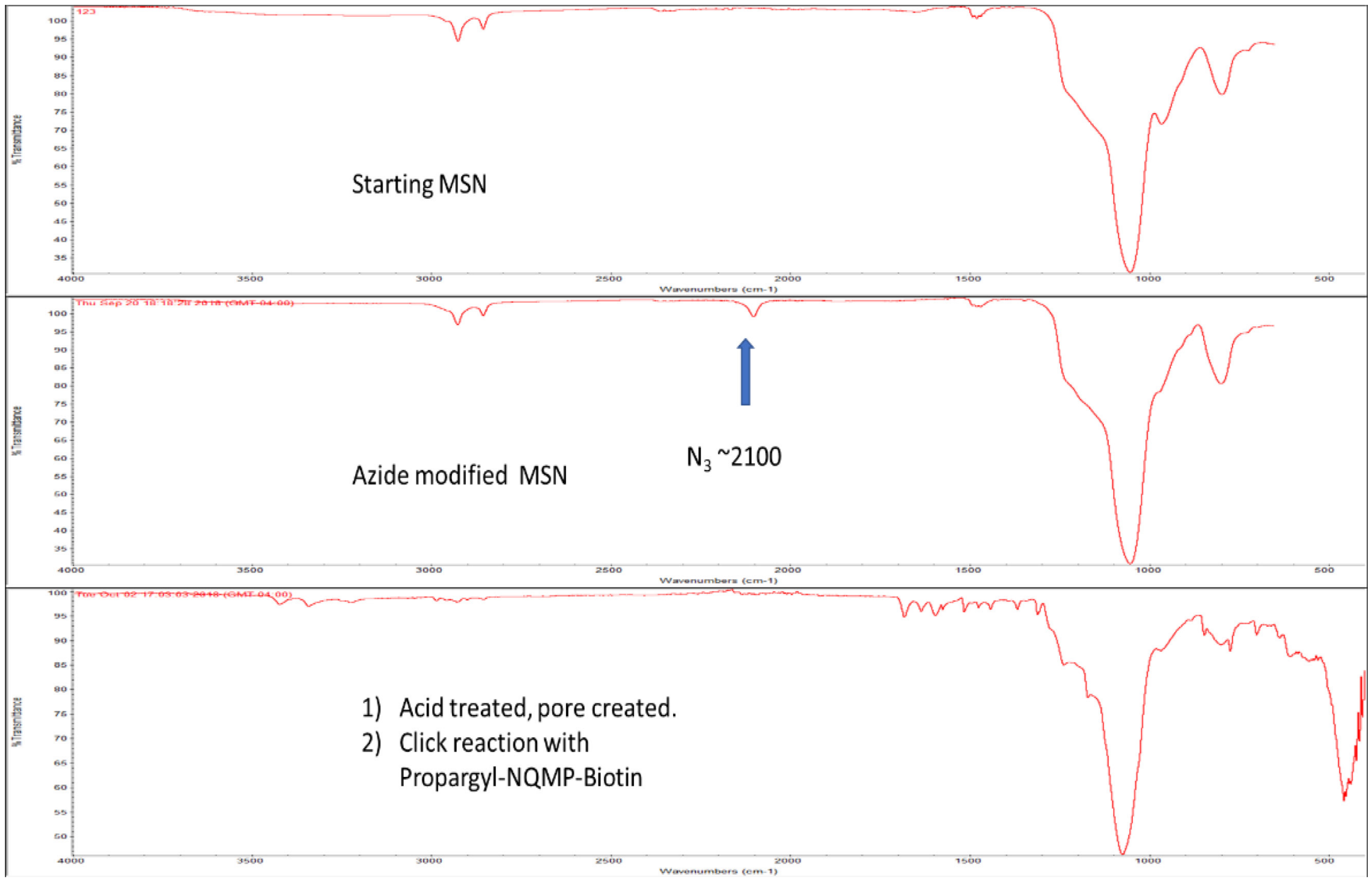

Figure S3. IR spectra for unmodified MSNPs (top), N_3-MSNPs (Middle), and **Si-NQMP-Biotin 1** (Bottom). The N_3-MSNPs spectrum clearly shows the azide stretching vibration band at ~ 2100 cm^{-1}. Upon CuAAC click reaction with **3d** the azide band disappears, while the carbonyl (~ 1700 cm^{-1}) and C=C aromatic (~1590 cm^{-1}) bands are clearly visible.

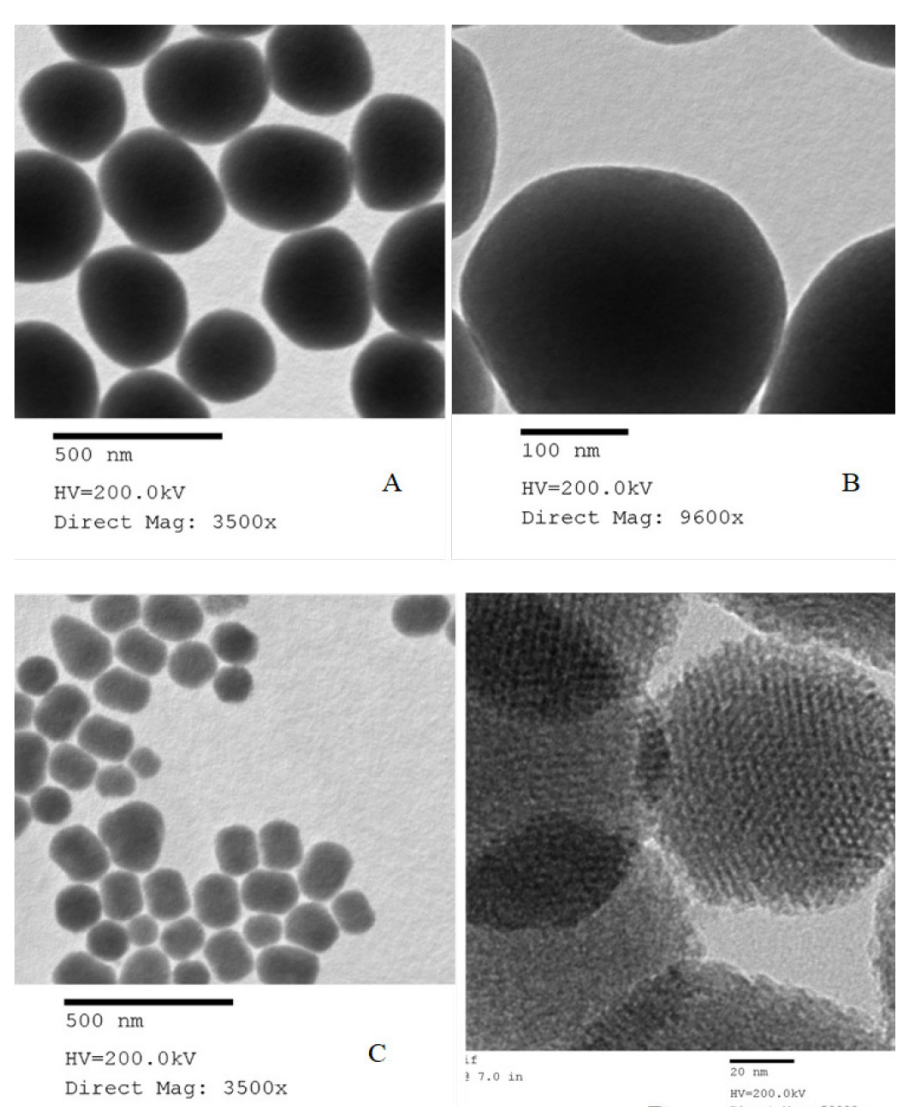

Figure S4. The TEM images of mesoporous silica nanoparticles. A) and B): crude particles (**MSNP**); C) and D): **NH_2-MSNP** and **N_3-MSNP** (both have identical TEM images)

Biotinylation of NH_2-MSNPs with carboxylate-NQMP-biotin (**3c**, Scheme 10). A mixture of NH_2-MSNs (50 mg), carboxylate-NQMP-biotin (**3c**, 50 mg, 0.072 mmol), EDC (21 mg, 0.11 mmol) and catalytic amounts of DMAP in 10 mL of DMF was stirred overnight at r.t. The resulting **Si-NQMP-Biotin 2** MNSPs were collected by centrifugation and washed with THF (2X10 mL), acetone (2X10 mL), and dried under reduced pressure overnight. The fluorescamine test showed complete conversion of amino-groups on the surface of MSNPs (Figure S2).

Biotinylation of N_3-MSNPs with propargyl-NQMP-biotin (**3d**, Scheme 10).

$BrCu(PPh_3)_3$ catalyst. A mixture of N_3-MSNs (50 mg), propargyl-NQMP-biotin (**3d**, 50 mg, 0.08 mmol) and $BrCu(PPh_3)_3$ (20 mg, 0.022 mmol) in 6 mL THF:TEA (1:1) was sonicated for 30 minutes till homogeneous suspension was formed. The reaction mixture was stirred at 60 ^{o}C for 12 h under argon. The resulting **Si-NQMP-Biotin 1** MNSPs were collected by centrifugation and washed with THF (2X10 mL), ethanol (2X10 mL), and dried under reduced pressure overnight. The IR spectrum of **Si-NQMP-Biotin 1** MNSPs showed the disappearance of azide band and the formation of carbonyl and aromatic C-C bond stretching vibration bands (Figure S3).

$CuSO_4$ catalyst. A mixture of N_3-MSNs (50 mg), propargyl-NQMP-biotin (**3d**, 50 mg) in 5 mL of DMF was sonicated for 30 minutes till homogeneous suspension was formed. 0.5 mL of a solution of $CuSO_4 \cdot 12H_2O$ (5.0 mg) and sodium ascorbate (16 mg) in DI water was added to the suspension and the reaction mixture was stirred at 70°C for 12 h under argon. The resulting **Si-NQMP-Biotin 1** MNSPs were collected by centrifugation and washed with DI water (2X10 mL), (2X10 mL), THF (2X10 mL), and ethanol (2X10 mL), then dried under reduced pressure for overnight.

Biotin concentration Determination using HABA Avidin kit (Pierce™ Biotin Quantitation Kit). 20 µL of the supernatant solution obtained after centrifugation of MSNPs was diluted with 160 µL of PBS, and 20 µL of HABA/Avidin stock solution was added to the mixture. The mixture was gently vortexed for 5 min and the absorbance at 500 nm was measured. The change in absorbance before and after irradiation was quantified using the calibration curve produced with standard PBS solutions containing from 0 to 20 µM of D-biotin.

Loading of Si-NQMP-Biotin particles with Doxorubicin (**DOX**). 5 mg of doxorubicin hydrochloride was added to the stirred suspension of 5 mg of **Si-NQMP-Biotin** MSNPs in 10 mL of aqueous buffer, sonicated for 20 minutes, and stirred 12 h. at r.t. in the dark. The MSNPs were collected by centrifugation and washed with 10 mL of DI water. The combined supernatant and the wash were diluted to 25 mL with DI water. Three 150 µL aliquots of the resulting solution were diluted to 3.0 mL with biphosphate buffer (pH = 7.4) and UV absorption at 480 nm was determined.

The concentration was calculated using 480 nm extinction coefficient of DOX = 11500 $M^{-1}cm^{-1}$. The amount of DOX remaining in the supernatant solutions was determined to be 3.44 mg when loading was conducted in biphosphate buffer (pH= 7.4) and 1.37 mg if loaded in bicarbonate buffer (pH= 9.3). These results correspond to loading capacity of pH 7.41 and pH 9.28 buffer are 0.31 mg and 0.73 mg DOX per 1 mg of **Si-NQMP-Biotin** MSNPs.

DOX concentration in the dialysis solution was determined using the same method.

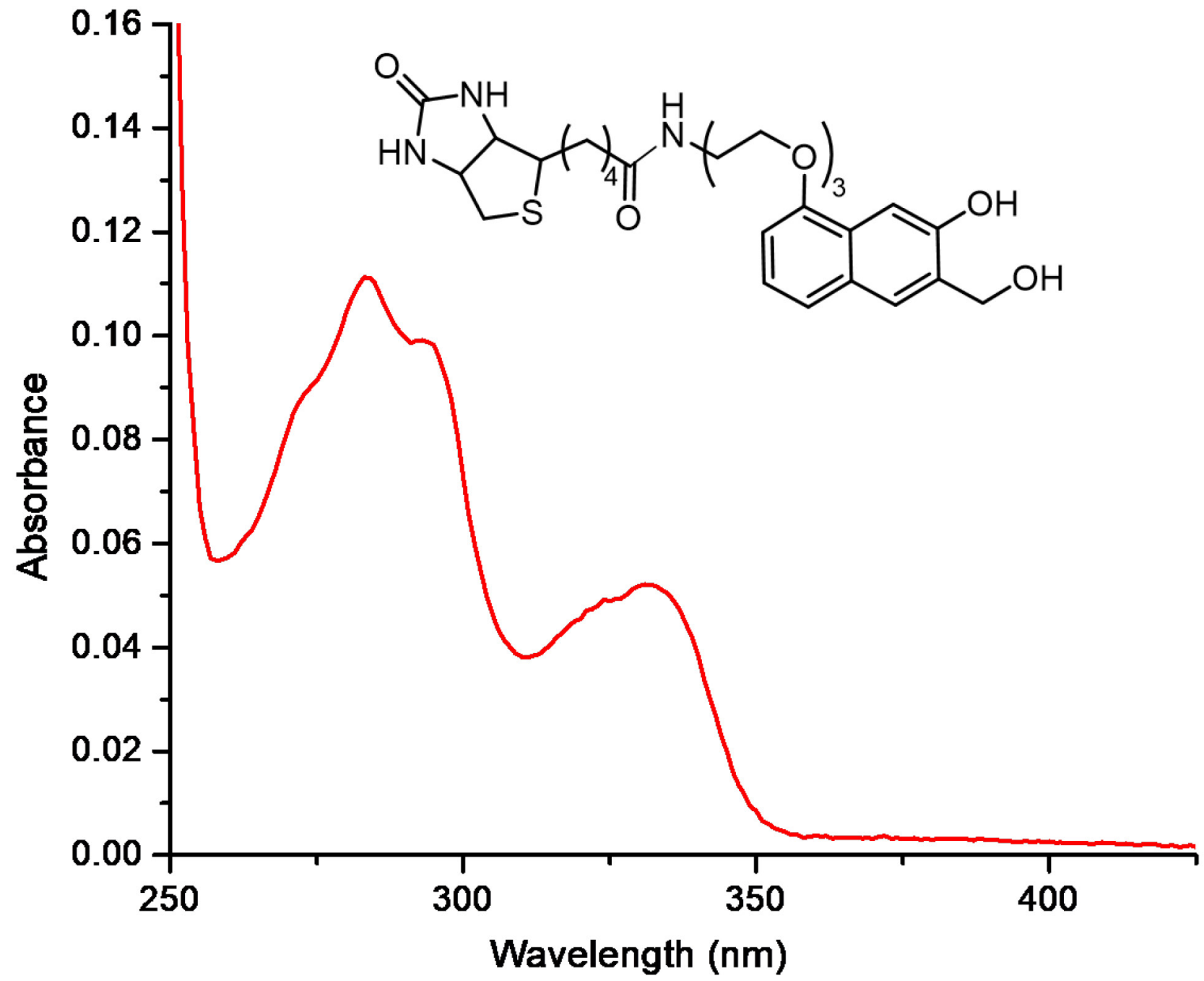

Figure S5. UV spectrum of 10 μM solution of NQMP-biotin in PBS.

Preparation of linkers

ADIBO carboxylic acid **15**,[3] 9-(azido-EG$_3$)-2, 2-dimethyl-4*H*-naphtho [2, 3-d][1,3] dioxine (**11c**, via the sequence **8a-9a-10-11a-11b-11c**)[4], ADIBO amine (**5**)[5] were prepared following previously reported procedures.

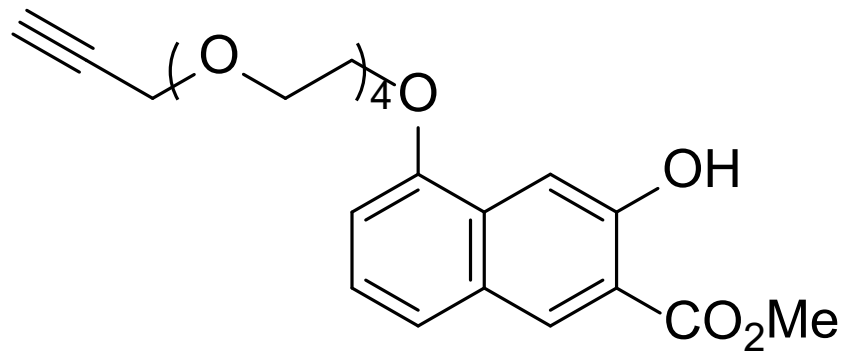

8b

Methyl 5-((3,6,9,12-tetraoxapentadec-14-yn-1-yl)oxy)-3-hydroxy-2-naphthoate (**8b**). Finely ground K_2CO_3 (0.80 g) was added to a solution of ester **7** (0.62 g, 2.9 mmol) in 25 mL of acetone, the mixture was brought to reflux, and a solution of Propargyl-EG$_4$-I (0.92 g, 2.6 mmol) in 5 mL of acetone was added slowly. The reaction was refluxed overnight and concentrated, the residue was extracted with 100 mL EtOAc, washed with water and brine, dried and purified using chromatography (EtOAc/Hexanes from 2:3 to 1:1) to give **8b** (0.88 g, 77%) as a bright yellow oil.

^{1}H NMR δ 10.41 (s, 1H), 8.41 (s, 1H), 7.73 (s, 1H), 7.37 (d, J = 8.4 Hz, 1H), 7.24 – 7.11 (t, J = 8.6 Hz, 1H), 6.81 (d, J = 7.4 Hz, 1H), 4.32 – 4.24 (m, 2H), 4.18 (d, J = 2.4 Hz, 2H), 4.00 (s, 5H), 3.85 – 3.77 (m, 2H), 3.68 (d, J = 2.7 Hz, 10H), 2.43 (s, 1H).

^{13}C NMR {^{1}H} δ 170.3, 156.1, 153.2, 131.8, 130.5, 128.0, 123.8, 121.4, 114.4, 107.3, 107.0, 79.7, 77.2, 74.5, 71.0, 70.7, 70.5, 69.8, 69.1, 68.0, 58.4, 52.6.

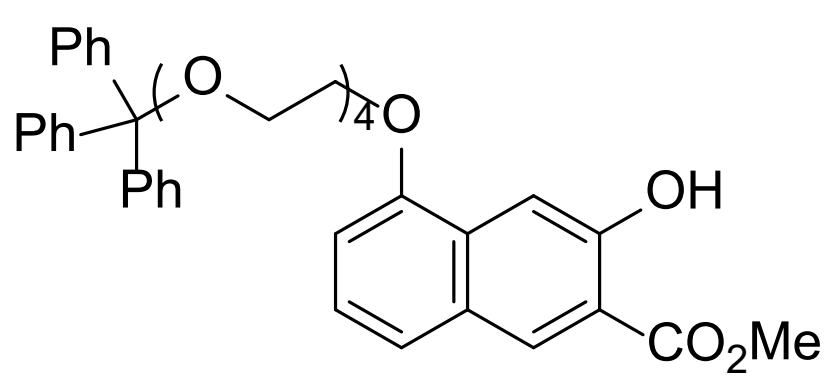

8c

Methyl 3-hydroxy-5-((1,1,1-triphenyl-2,5,8,11-tetraoxatridecan-13-yl)oxy)-2-naphthoate (**8c**) A solution of Trityl-EG$_4$-I ($Ph_3C(OCH_2CH_2)_4I$, 4.50 g, 8.24 mmol) in 100 mL of acetone, and 3.6 g of potassium carbonate were added to a solution of **7** (1.80 g, 8.24 mmol) and the reaction mixture was refluxed for 12 h, cooled down to r.t., filtered, and concentrated under reduced pressure. The residue was dissolved in EtOAc (200 mL), washed with water and brine, concentrated under reduced pressure, and purified by chromatography (EtOAc:Hexanes 1:3), to give 2.1 g (40%) of **8c** as a brownish solid.

^{1}H NMR δ 10.41 (s, 1H), 8.41 (s, 1H), 7.74 (s, 1H), 7.46 (d, J = 7.9 Hz, 6H), 7.37 (d, J = 8.8 Hz, 1H), 7.28-7.09 (m, 10H), 6.76 (d, J = 7.6 Hz, 1H), 4.30 – 4.14 (m, 2H), 3.99 – 3.87 (m, 5H), 3.80 – 3.60 (m, 10H), 3.23 (t, J = 5.2 Hz, 2H).

^{13}C{^{1}H} NMR δ 170.3, 156.1, 153.2, 144.1, 131.8, 130.5, 128.7, 127.8, 126.9, 123.8, 121.4, 114.3, 107.24, 107.0, 86.5, 77.4, 77.1, 76.7, 71.0, 70.7, 69.7, 67.9, 63.3, 52.5.

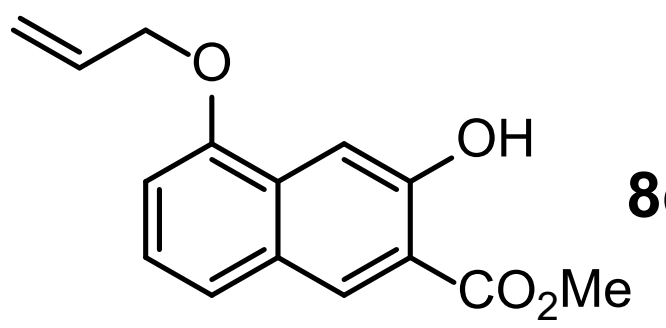

8d

Methyl 5-(allyloxy)-3-hydroxy-2-naphthoate (**8d**). K_2CO_3 (6.54 g, 47.4 mmol) was added to a solution of ester **7** (5.17 g, 23.7 mmol) in 100mL of acetone. The reaction mixture was heated to reflux while being stirred for 30 min, then allyl bromide (2.87 g, 23.7 mmol) was added, and the reaction was refluxed for 12 h. The solvent was removed under reduced pressure, EtOAc and 1 M HCl (200 mL each) were added and the organic layer was separated. The organic layer was washed with water and brine, dried over Na_2SO_4, and concentrated under reduced pressure. The product was purified by chromatography (15% EtOAc in Hexanes), to produce 4.75 g (78%) of **8d** as a light-yellow solid.

^{1}H NMR δ 10.33 (s, 1H), 8.36 (s, 1H), 7.69 (s, 1H), 7.31 (d, J = 8.4 Hz, 1H), 7.13 (t, J = 4.0 Hz, 1H), 6.74 (d, J = 7.6 Hz, 1H), 6.08 (m, 1H), 5.45 (dd, J = 17.3, 1.5 Hz, 1H), 5.26 (dd, J = 10.6, 1.2 Hz, 1H), 4.71 – 4.54 (d, J=5.2 Hz, 2H), 3.94 (s, 3H).

^{13}C NMR {^{1}H} δ 170.3, 156.2, 153.0, 130.0, 131.9, 130.6, 128.0, 123.8, 121.4, 117.6, 114.4, 107.4, 107.0, 69.0, 52.6.

ESI MS, *m/z*: $[M+Na]^+$ Calcd for $C_{15}H_{14}NaO_4^+$ 281.2408; Found 281.0

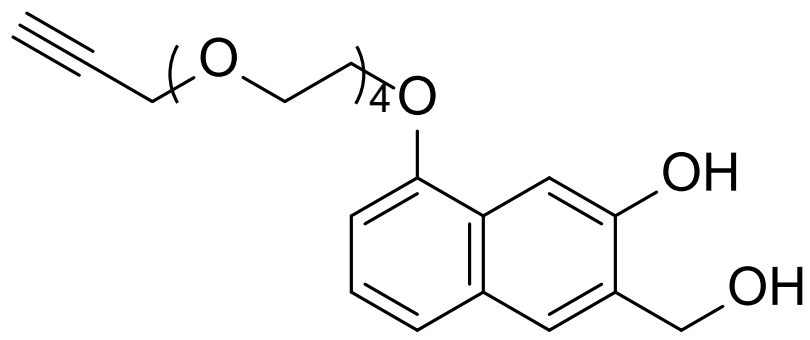

9b

r **8-((3,6,9,12-Tetraoxapentadec-14-yn-1-yl)oxy)-3-(hydroxymethyl)naphthalen -2-ol** (**9b**)**:** To a suspension of LAH (1.3 mL, 2.4 M in THF, 3.1 mmol) in 40 mL THF at 0°C, a solution of **8b** (0.88 g, 2.0 mmol) in 10 mL THF was added dropwise. The reaction was warmed up to room temperature and stirred for 5 h. Then 15 mL of 1 M HCl was added dropwise, stirred for 10 min, and separated between 150 mL EtOAc and 150 mL water. The organic layer was washed by brine

and dried, concentrated, and purified by chromatography (75% EtOAc in Hexanes), yielding **9b** (0.78 g, 90%) as a light-yellow oil.

^{1}H NMR δ 7.53 (s, 1H), 7.39 (s, 1H), 7.17 (d, J = 8.4 Hz, 1H), 7.04 (s, 1H), 6.59 (d, J = 7.5 Hz, 1H), 4.76 (s, 2H), 4.08 (m, 2H), 4.04 (d, J = 2.4 Hz, 2H), 3.77 (d, J = 4.7 Hz, 2H), 3.66 – 3.47 (m, 12H), 2.33 (s, 1H).

^{13}C{^{1}H} NMR δ 153.5, 153.3, 129.3, 128.7, 126.9, 126.8, 123.2, 120.5, 105.9, 105.1, 79.5, 74.9, 70.7, 70.6, 70.5, 70.3, 69.8, 69.0, 68.3, 63.3, 60.5, 58.3.

MALDI-TOF MS, *m/z*: $[M-H]^-$ Calcd for $C_{22}H_{27}O_7^-$ 403.18; Found 403.2.

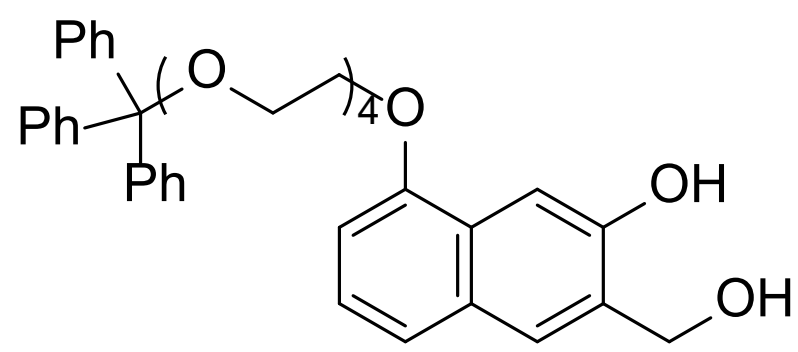

9c

3-(Ethoxymethoxy)-5-((1,1,1-triphenyl-2,5,8,11-tetraoxatridecan-13-yl)oxy)naphthalen-2-ol (**9c**). A solution of **8c** (1.25 g, 1.8 mmol) in 10 mL of THF was slowly added to a suspension of LAH (0.28 g, 7.2 mmol) in 20 mL of THF at 0°C. The stirred reaction mixture was allowed to warm up to r.t. and stirred overnight. EtOAc and water (50 mL each) were added, the organic layer was separated, washed with water and brine, dried over Na_2SO_4, and concentrated under reduced pressure. The product was purified by chromatography (EtOAc : Hexanes 1:2) to produce 1.1 g (95%) of **9c** as a clear oil.

^{1}H NMR δ 7.65 (s, 1H), 7.49 (d, J = 13.9 Hz, 1H), 7.45 (d, J = 8.1 Hz, 6H), 7.31 – 7.12 (m, 11H), 6.72 (d, J = 7.5 Hz, 1H), 4.81 (d, J = 4.5 Hz, 2H), 4.18 (t, J = 12.9 Hz, 2H), 3.85 (dd, J = 15.3, 11.2 Hz, 2H), 3.72 (dt, J = 9.6, 4.8 Hz, 10H), 3.23 (dd, J = 20.7, 15.7 Hz, 2H).

^{13}C{^{1}H} NMR δ 153.5, 144.0, 129.4, 129.0, 128.7, 127.8, 127.0, 123.2, 120.6, 106.4, 105.0, 86.7, 76.8, 70.82, 70.7, 70.6, 69.8, 68.4, 63.5, 63.2.

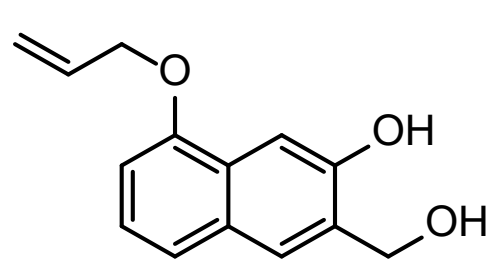

3e

8-(Allyloxy)-3-(hydroxymethyl)naphthalen-2-ol (**3e**). LAH solution (2.4 M in THF, 11.5mL, 27.6 mmol) was added dropwise to a solution of ester **8d** (4.75 g, 18.4 mmol) in 150mL anhydrous THF cooled by the ice bath. The reaction was stirred for 3 h at r.t. and quenched by dropwise addition of water until no gas was produced. EtOAc and 1 M HCl (200 mL each) were added and the organic layer was separated. The organic layer was washed with water and

brine, dried over Na_2SO_4, and concentrated under reduced pressure. The product was purified by chromatography (50% EtOAc in Hexanes) to give 3.98 g (94%) of **3e** as a light-yellow oil.

^{1}H NMR : δ 7.69 (s, 1H), 7.54 (s, 1H), 7.32 (d, J = 8.4 Hz, 1H), 7.22 (t, J = 8.0 Hz, 1H), 7.07 (s, 1H), 6.79 (d, J = 7.6 Hz, 1H), 6.22 – 6.12 (m, 1H), 5.55 (dd, J = 17.2, 1.6 Hz, 1H), 5.36 (dd, J = 10.4, 1.2 Hz, 1H), 4.99 (d, J = 4.4 Hz, 2H), 4.69 (d, J = 5.2 Hz, 2H), 2.40 (s, 1H).

^{13}C NMR {^{1}H} δ 153.26, 133.34, 129.48, 127.60, 126.94, 126.81, 123.59, 120.14, 117.40, 105.98, 105.62, 68.96, 64.51.

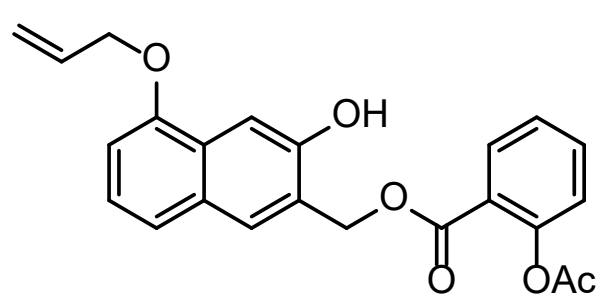

6

(5-(Allyloxy)-3-hydroxynaphthalen-2-yl)methyl 2-acetoxybenzoate (**6**, **NQMP-ASA**). EDC hydrochloride (0.55 g, 2.9 mmol) and a catalytic amount of DMAP were added to a stirred solution of aspirin (0.47 g, 2.6 mmol) in 10 mL of DMF. The mixture was stirred for 15 min and added to a solution of **3e** (0.67 g, 2.9 mmol) in 10 mL of DMF. The reaction mixture was stirred for 24 h at r.t. and quenched by adding EtOAc and water (50 mL each). The organic layer was separated, washed with brine, dried over Na_2SO_4, concentrated under reduced pressure, and the product purified by chromatography (20% EtOAc in Hexanes), to yield 380 mg (39%) of **6** as yellow oil.

^{1}H NMR δ 8.05 (dd, J = 7.6, 1.6 Hz, 1H), 7.81 (s, 1H), 7.71 (s, 1H), 7.51 – 7.47 (m, 1H), 7.28 (d, J = 8, 1H), 7.23 (t, J = 7.6 Hz, 1H), 7.15 (t, J = 8.0 Hz, 1H), 7.09 (d, J = 8.1 Hz, 1H), 6.87 (s, 1H), 6.78 (d, J = 7.6 Hz, 1H), 6.08 (m, 1H), 5.53 – 5.47 (m, 3H), 5.32 – 5.15 (d, J = 12, 1H), 4.68 (t, J = 5.2 Hz, 2H), 2.22 (s, 3H).

^{13}C NMR {^{1}H} δ 170.1,163.6, 154.0, 151.3, 146.1, 134.9, 133.1, 132.7, 132.3, 128.2, 126.4, 126.2, 125.7, 124.1, 122.3, 120.2, 117.7, 114.7, 105.6, 69.1, 60.8, 21.1

Preparation of Si-NQMP-ASA MSNPs (Scheme 8).

(3-Hydroxy-5-(3-(triethoxysilyl)propoxy)naphthalen-2-yl)methyl 2-acetoxybenzoate (6-TEOS). Triethoxysilane (180 μL, 0.9 mmol) and 30 μL of Karstedt's catalyst were added to a solution of to **6** (160 mg, 0.43 mmol) in 10 mL THF, the reaction mixture was stirred for 12 h at 60 °C. The solution was filtered to remove the solid phase, the filtrate was concentrated, and purified by chromatography (25% EtOAc in Hexanes) to yield product as a clear oil, which was used in the next step without further purification.

A mixture of 100 mg MSN and 100 mg Asp-NQMP-TEOS in 20mL anhydrous toluene was sonicated for 20 min until a clear solution formed and then refluxed for 24 h with stirring. The resulting suspension was cooled down to room temperature, particles were collected by centrifuge (8000 rpm, 10 min), washed twice with water and methanol, and dried under reduced pressure.

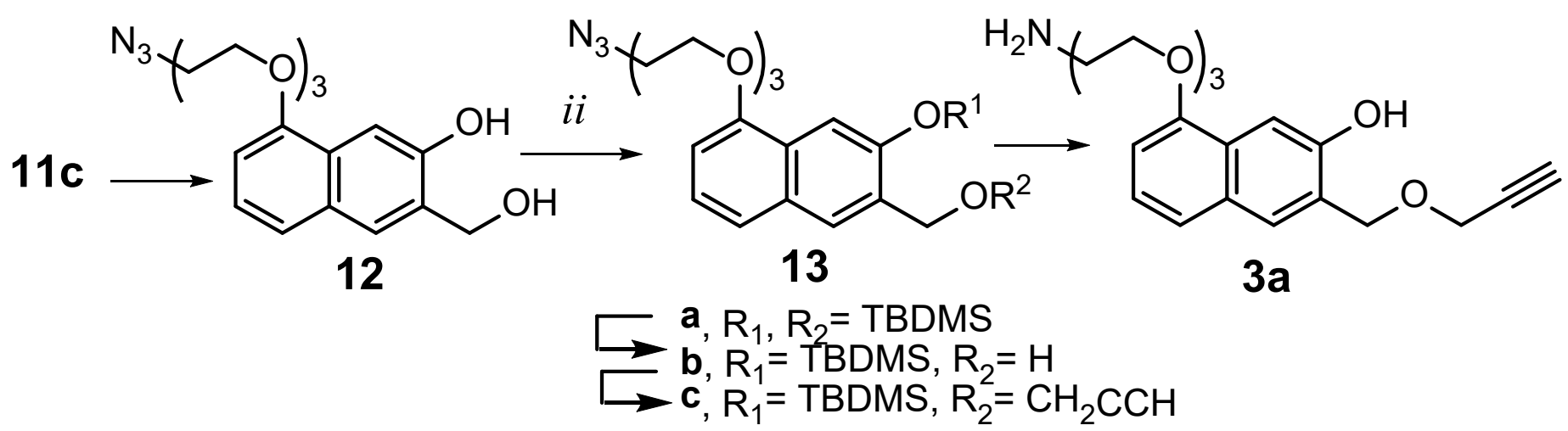

Synthesis of Compound 13a. Amberlyst-15 resin (~1.2 g) was added to a solution of **11c**[4] (1.32 g, 3.4 mmol) in 50 mL of methanol and stirred for 2 h at r.t. The resin was removed by filtration through a cotton plug, and the methanol solution was passed through a short silica gel column to yield 1.14 g (96%) of **12** as pale-yellow oil. Compound **12** was used in the next step without purification.

tert-Butyldimethylsilyl chloride (1.30 g, 8.64 mmol), imidazole (1.17 g. 17.28 mmol), and a catalytic amount of DMAP were added to a solution of **12** (1 g, 2.88 mmol) in 10 mL of anhydrous DMF and the reaction mixture was stirred overnight at r.t. The reaction mixture was poured into 100 mL of saturated sodium bicarbonate solution, and the product **13a** was extracted with ethyl acetate (3 x 100 mL), the organic layer was washed with brine, dried over sodium sulfate, the solvent was removed under reduced pressure, and the product **13a** was purified by chromatography (ether : DCM 1:3) to yield 1.33 g of **13a** (80 %).

^{1}H NMR δ 7.84 (s, 1H), 7.53 (s, 1H), 7.39 (d, J = 8.2 Hz, 1H), 7.22 (t, J = 8.0 Hz, 1H), 6.74 (d, J = 7.6 Hz, 1H), 4.89 (s, 2H), 4.29 (t, J = 4.8 Hz, 2H), 4.01 (dd, J = 5.6, 4.1 Hz, 2H), 3.89 – 3.79 (m, 2H), 3.73 – 3.64 (m, 4H), 3.37 (t, J = 5.1 Hz, 2H), 1.04 (s, 9H), 0.99 (s, 9H), 0.29 (s, 6H), 0.14 (s, 6H).

^{13}C{^{1}H} NMR δ 153.6, 150.8, 134.2, 130.4, 125.6, 125.3, 123.6, 120.6, 107.6, 104.9, 71.4, 71.1, 70.31, 70.30, 68.3, 61.4, 50.9, 26.3, 26.0, 18.8, 18.5, -4.04, -5.09.

HRMS (ESI/ Orbitrap), *m/z*: $[M+H]^+$ Calcd for $C_{29}H_{50}N_3O_5Si_2^+$ 576.3284; Found 576.3279

Synthesis of Compound 13b. $CeCl_3.7H_2O$ (1.71 g. 4.62 mmol) was added to a solution of **13a** (1.3 g, 2.31 mmol) in 75mL of anhydrous MeCN, the reaction mixture was refluxed for 4 h, and stirred overnight at r.t. The reaction mixture was poured into 200 mL of saturated solution of

sodium bicarbonate, extracted with ethyl acetate (3x150); the organic layer was washed with brine, dried over sodium sulfate, the solvent was removed under reduced pressure, and the product **13b** was purified by chromatography (ether:DCM 2:3) to yield 0.89 g of **13b** (85 %).

^{1}H NMR (400 MHz, Chloroform-d) δ 7.74 (s, 1H), 7.61 (s, 1H), 7.36 (d, J = 8.2 Hz, 1H), 7.23 (t, J = 7.9 Hz, 1H), 6.76 (dd, J = 7.7, 0.9 Hz, 1H), 4.83 (s, 2H), 4.29 (t, J = 4.8 Hz, 2H), 4.01 (dd, J = 5.5, 4.1 Hz, 2H), 3.87 – 3.77 (m, 2H), 3.77 – 3.58 (m, 4H), 3.37 (t, J = 5.1 Hz, 2H), 1.07 (s, 9H), 0.35 (s, 6H).

$^{13}C\{^{1}H\}$ NMR δ 153.5, 151.5, 133.3, 130.2, 127.0, 126.1, 124.0, 120.4, 108.2, 105.3, 71.26, 71.0, 70.20, 70.17, 68.1, 62.3, 50.8, 26.0, 18.4, -4.1.

HRMS (ESI/ Orbitrap), *m/z*: $[M+H]^+$ Calcd $C_{23}H_{36}N_3O_5Si^+$ 462.2419; Found 462.2414

Synthesis of Compound 13c. NaH (52 mg, 2.24 mmol) was added to a solution of compound **13b** (850 mg, 1.84 mmol) in 10mL of THF and the resulting suspension was stirred at 0 °C for 30 min, followed by the dropwise addition of propargyl bromide (260 mg, 2.24 mmol). The reaction mixture was allowed to warm up to room temperature, stirred for 4 h at r.t., quenched with saturated ammonium chloride, and the product was extracted with ether (3x150). The combined organic layers were washed with brine solution, dried over anhydrous sodium sulfate, and the solvent was removed under reduced pressure. The resulting residue was purified by chromatography (ethyl acetate:hexanes 1:9) to yield 745 mg (81%) of compound **13c**.

^{1}H NMR δ 7.71 (s, 1H), 7.46 (s, 1H), 7.24 (d, J = 8.1 Hz, 1H), 7.09 (t, J = 7.9 Hz, 1H), 6.63 (d, J = 7.6 Hz, 1H), 4.74 (s, 2H), 4.68 (d, J = 2.5 Hz, 2H), 4.15 (t, J = 4.9 Hz, 2H), 3.85 (t, J = 4.9 Hz, 2H), 3.70 – 3.63 (m, 2H), 3.50 (dd, J = 5.7, 3.8 Hz, 2H), 3.34 (t, J = 5.2 Hz, 2H), 2.68 (t, J = 5.1 Hz, 2H), 2.41 (t, J = 2.4 Hz, 1H), 0.83 (s, 9H), 0.00 (s, 6H).

$^{13}C\{^{1}H\}$ NMR δ 153.8, 152.9, 132.1, 130.4, 125.7, 125.4, 123.9, 120.6, 105.5, 101.0, 78.8, 75.7, 73.6, 71.2, 70.6, 70.1, 68.3, 60.7, 56.1, 41.9, 26.2, 18.7, -5.1.

HRMS (ESI/ Orbitrap), *m/z*: $[M+H]^+$ Calcd for $C_{26}H_{38}N_3O_5Si^+$ 500.2575; Found 500.2579.

Synthesis of photolabile linker 3a. A solution of **13c** (0.7g, 1.4 mmol) in ether (30 mL) was added to a stirred suspension of $LiAlH_4$ (106 mg, 2.8 mmol) in anhydrous THF at 0 ^{0}C, stirred for 4 h at r.t., quenched with 5% HCl, neutralized with $NaHCO_3$ and extracted with ether. The ether solution was washed with brine, dried over anhydrous magnesium sulfate, and the solvent was removed under reduced pressure to yield 470 mg (94%) of amine **3a** as a yellow oil.

^{1}H NMR δ 7.74 (s, 1H), 7.67 (s, 1H), 7.38 (d, J = 8.2 Hz, 1H), 7.28 (t, J = 7.9 Hz, 1H), 6.83 (d, J = 7.6 Hz, 1H), 4.88 (d, J = 2.4 Hz, 2H), 4.85 (s, 2H), 4.33 (t, J = 4.9 Hz, 2H), 4.02 (dd, J = 5.7, 4.1 Hz, 2H), 3.86 – 3.80 (m, 2H), 3.70 – 3.64 (m, 2H), 3.54 – 3.47 (m, 2H), 2.84 (t, J = 5.2 Hz, 2H), 2.59 (t, J = 2.4 Hz, 1H).

$^{13}C\{^{1}H\}$ NMR δ 153.6, 153.5, 131.1, 130.1, 127.4, 125.9, 124.2, 120.4, 105.9, 101.6, 78.3, 75.8, 73.6, 71.0, 70.5, 70.0, 68.2, 62.0, 56.1, 41.8.

HRMS (ESI/ Orbitrap), *m/z*: $[M+H]^+$ Calcd for $C_{20}H_{26}NO_5{}^+$ 360.1805; Found 360.1808.

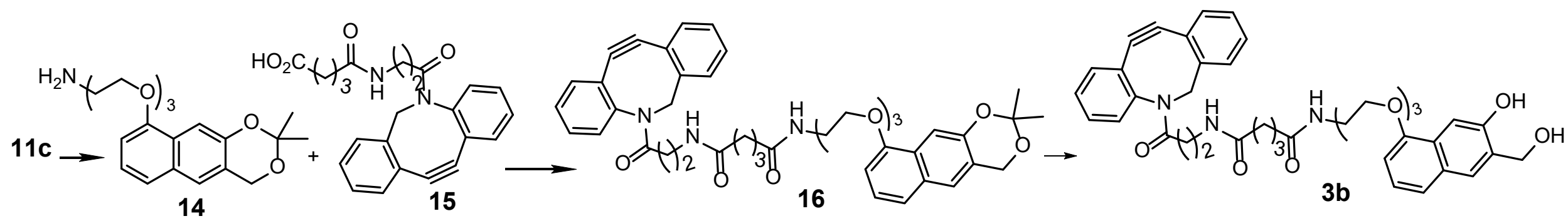

9-(Amino-TEG)-2,2-dimethyl-4H-naphtho[2,3-d][1,3]dioxine (**14**): **11c** (0.2 g, 0.52 mmol) was added to a suspension of $LiAlH_4$ (0.038g, 1 mmol) in anhydrous ethyl ether at 0^0C. The reaction mixture is warmed to r.t., stirred for 4 h, quenched with 5% HCl, and extracted in ethyl ether. The organic layer was washed with brine, dried over anhydrous magnesium sulfate, and the solvent was removed under reduced pressure to yield 152 mg (81%) of the amine **14**, which was used in the next step without further purification.

^{1}H NMR (CD_3CN) δ 7.51 (s, 1H), 7.49 (m, 2H), 7.33 (d,8 Hz, 1H), 7.23 (t, 7.6 Hz, 1H), 6.80 (d, 8 Hz 1H), 5.03 (s, 2H), 4.20-4.22 (m, 2H), 3.88-3.90 (m, 2H), 3.68-3.70 (m, 2H), 3.59-3.60 (m, 2H), 3.41-3.43 (m, 2H), 2.73 (br s, 2H), 2.10 (br s, 2H), 1.55 (s, 6H).

$^{13}C\{^{1}H\}$ NMR (CD_3CN) δ 26.1, 41.6, 62.5, 69.8, 71.8, 72.2, 72.3, 101.7, 106.8, 107.9, 121.6, 123.8, 125.3, 125.6, 127.7, 131.3, 151.4, 156.2.

HRMS (EI Orbitrap), *m/z*: $[M]^+$ Calcd for $C_{20}H_{27}NO_5$ 361.1889; Found 361.1889.

Synthesis of Compound 16: 1-Ethyl-3-(3-dimethylaminopropyl)carbodiimide (140 mg, 0.9 mmol) and catalytic amount of DMAP were added to a solution of ADIBO-carboxylic acid (**15**) (450 mg, 1.14 mmol) in 8 mL of anhydrous DMF, followed by a dropwise addition of a solution of 9-(Amino-TEG)-2, 2-dimethyl-4*H*-naphtho [2, 3-d][1,3] dioxine (**14**) (456 mg, 1.26 mmol) in 2 mL of DMF. The mixture was stirred for 12 h at r.t., solvent was removed in vacuum, the residue was dissolved in DCM, washed with $NaHCO_3$ solution and brine, dried over anhydrous magnesium sulfate, and concentrated under reduced pressure. The residue was purified by chromatography

(10% MeOH in DCM) to yield 715 mg (85%) of compound **16**.

^{1}H NMR δ 7.60 (d, J = 7.5 Hz, 1H), 7.46 – 7.29 (m, 5H), 7.14 (t, J = 7.9 Hz, 1H), 6.66 (d, J = 7.5 Hz, 1H), 6.54 (t, J = 5.6 Hz, 1H), 6.41 (t, J = 6.0 Hz, 1H), 5.06 (d, J = 13.8 Hz, 1H), 5.02 (s, 2H), 4.19 (t, J = 4.8 Hz, 1H), 3.89 (t, J = 4.7 Hz, 2H), 3.71 (t, J = 4.5 Hz, 2H), 3.60-3.63 (m, 3H), 3.51-3.59 (m, 3H), 3.10-3.50 (m, 3H), 3.04 (t, J = 4.8 Hz, 2H), 2.60 – 2.34 (m, 1H), 2.16 – 1.66 (m, 6H), 1.55 (s, 6H).

^{13}C{^{1}H} NMR δ 172.7, 172.6, 172.5, 153.7, 151.2, 149.7, 148.2, 132.3, 129.7, 129.2, 128.8, 128.6, 128.4, 128.1, 127.4, 126.3, 125.8, 123.9, 123.4, 123.1, 122.7, 121.8, 120.2, 115.0, 107.9, 107.2, 104.9, 100.0, 71.0, 70.4, 70.0, 69.9, 68.0, 61.3, 55.8, 39.4, 35.6, 35.3, 35.2, 34.6, 25.2, 22.1.

HRMS (EI Orbitrap), *m/z*: $[M+H]^+$ Calcd for $C_{43}H_{48}N_3O_8{}^+$ 734.3436; Found 734.3431.

Photolabile linker 3b. Amberlyst-15 resin (0.33 g) was added to a solution of **16** (0.7 g, 0.95 mmol) in 50 mL of methanol and stirred for 2 h at r.t. The resin was removed by filtration through a cotton plug and the methanol solution was passed through a short silica gel column to yield 0.62 g (93%) of **3b** as a pale yellow oil.

^{1}H NMR δ 7.72 (d, J = 7.4 Hz, 2H), 7.50 – 7.28 (m, 8H), 7.25 (t, J = 7.9 Hz, 1H), 6.78 (d, J = 7.5 Hz, 1H), 6.65 (t, J = 5.5 Hz, 1H), 6.52 (t, J = 6.0 Hz, 1H), 5.18 (d, J = 13.9 Hz, 1H), 5.12 (s, 2H), 4.26 (t, J = 4.8 Hz, 2H), 3.96 (t, J = 4.8 Hz, 2H), 3.77 (t, J = 4.8 Hz, 2H), 3.69 (m, 3H), 3.61 (ddd, J = 6.1, 4.3, 1.4 Hz, 2H), 3.53 – 3.14 (m, 3H), 3.12 (t, J = 4.8 Hz, 2H), 2.60 – 2.49 (m, 1H), 2.25 – 1.77 (m, 6H).

^{13}C{^{1}H} NMR δ 172.6, 172.4, 172.3, 153.5, 151.0, 149.5, 148.1, 132.1, 129.5, 129.1, 128.6, 128.4, 128.3, 127.9, 127.3, 126.2, 125.6, 123.7, 123.2, 123.0, 122.57, 121.60, 120.0, 114.8, 107.8, 107.0, 104.8, 99.9, 70.9, 70.3, 69.8, 69.7, 67.9, 61.2, 55.6, 39.2, 35.4, 35.1, 35.0, 34.5, 21.9.

HRMS (EI Orbitrap), *m/z*: $[M+H]^+$ Calcd for $C_{40}H_{44}N_3O_8{}^+$ 694.3123; Found 694.3126

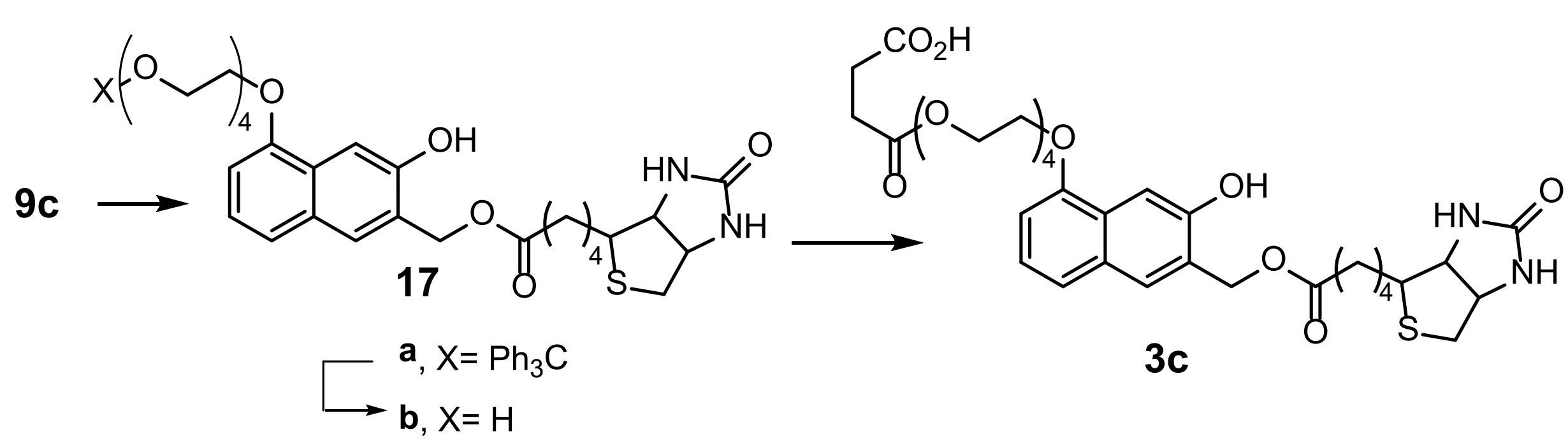

(3-Hydroxy-5-((1,1,1-triphenyl-2,5,8,11-tetraoxatridecan-13-yl)oxy) naphthalen-2-yl)methyl biotinylate (17a). A suspension of *D*-biotin (1.2 g, 5.0 mmol), EDC (0.90 g, 5.8 mmol), and catalytic amount of DMAP in 20 mL of anhydrous DMF was stirred for 15 min at r.t. A solution of **9c** (2.0 g, 3.3 mmol) in 5 mL DMF was slowly added to the mixture, stirred for 8 h, diluted with 100 mL of EtOAc (white precipitate was formed), washed with 100 mL water and 100 mL brine, organic layer separated, dried over Na_2SO_4, and concentrated under reduced pressure. The product was purified by chromatography (10% MeOH in DCM) to give 1.95 g (71%) of **17a** as colorless crystals and 0.8 g (22%) of disubstituted product as a clear oil.

^{1}H NMR δ 9.01 (s, 1H), 7.72 (d, J = 18.9 Hz, 2H), 7.46 (d, J = 7.4 Hz, 6H), 7.35 (d, J = 7.6 Hz, 1H), 7.32 – 7.14 (m, 10H), 6.75 (d, J = 7.6 Hz, 1H), 5.47 (s, 1H), 5.31(d, J = 11.7 Hz, 1H), 5.15 (d, J = 11.8 Hz, 1H), 4.34 – 4.20 (m, 3H), 4.05 (m, 1H), 3.87 – 3.80 (m, 2H), 3.80 – 3.64 (m, 10H), 3.29 – 3.19 (m, 2H), 2.99 (m, 2H), 2.75 (dd, J = 12.8, 4.9 Hz, 1H), 2.59 (d, J = 12.9 Hz, 1H), 2.47 – 2.30 (m, 3H), 1.81–1.58 (m, 4H), 1.43 – 1.30 (m, 2H).

^{13}C{^{1}H} NMR δ 174.1, 163.9, 153.3, 144.1, 130.2, 129.2, 128.4, 127.8, 127.4, 127.0, 124.9, 123.2, 120.5, 110.3, 92.1, 75.1, 74.8, 74.0, 73.2, 72.4, 68.1, 66.7, 66.2, 65.0, 60.4, 44.3, 32.8, 29.2

(3-hydroxy-5-(2-(2-(2-(2-hydroxyethoxy)ethoxy)ethoxy)ethoxy)naphthalen-2-yl)methyl biotinylate (**17b**). 10 mL of 4% TFA solution in DCM were added dropwise to a solution of **17a** (0.70 g, 0.84 mmol) in 10 mL of anhydrous DCM. The reaction was stirred at r.t. for 10 min and quenched with 20 mL of concentrated $NaHCO_3$ solution. The DCM layer was separated out and dried with Na_2SO_4, concentrated, and purified by chromatography (10% MeOH in DCM) to give 0.47 g (94%) of **17b** as a white solid.

^{1}H NMR (CD_3OD) δ 9.30 (s, 1H), 7.71 (s, 1H), 7.64 (s, 1H), 7.34 (d, J = 8.2 Hz, 1H), 7.16 (t, J = 7.9 Hz, 1H), 6.74 (d, J = 7.6 Hz, 1H), 6.08 (s, 1H), 5.53 (s, 1H), 5.35 (d, J = 12.1 Hz, 1H), 5.24 (d, J = 12.1 Hz, 1H), 4.60 (s, 1H), 4.34 – 4.22 (m, 3H), 4.08 (dd, J = 7.9, 4.5 Hz, 1H), 3.92 (dd, J = 5.6, 3.1 Hz, 2H), 3.83 – 3.67 (m, 10H), 3.67 – 3.60 (m, 2H), 3.47 (s, 1H), 3.08 (s, 1H), 2.95 (t, J = 7.3 Hz, 1H), 2.68 (dd, J = 12.9, 4.8 Hz, 1H), 2.57 (d, J = 12.9 Hz, 1H), 2.38 (td, J = 7.4, 3.7 Hz, 2H), 1.72 – 1.50 (m, 4H), 1.42 – 1.27 (m, J = 6.6 Hz, 2H).

^{13}C{^{1}H} NMR (CD_3OD) δ 178.3, 166.9, 157.4, 134.1, 132.2, 131.4, 131.0, 130.5, 128.2, 125.1, 123.2, 120.5, 106.4, 105.3, 86.6, 71.0, 70.8,70.1, 68.2, 63.3, 63.0, 61.1, 60.2, 55.2, 40.3, 33.6, 27.8, 24.6

1-((7-Hydroxy-6-(biotinylate)methyl)naphthalen-1-yl)oxy)-3,6,9,12-tetraoxapentadecan-15-oic acid (**3c**). 1mL TEA, 0.30 g succinic anhydride were added sequentially to a solution of **17b**

(0.47 g, 0.80 mmol) in 25 mL of anhydrous DCM, the reaction was stirred 12 h, and quenched with 50 mL of 1 M aqueous HCl. The DCM layer was separated, dried over Na_2SO_4, concentrated under reduced pressure, and purified by chromatography (10% MeOH in DCM) to give **3c** (0.44 g, 80%) as a clear oil.

^{1}H NMR δ 7.72 (s, 1H), 7.66 (s, 1H), 7.34 (d, J = 8.2 Hz, 1H), 7.17 (t, J = 7.9 Hz, 1H), 6.76 (d, J = 7.6 Hz, 1H), 6.56 (s, 1H), 6.04 (s, 1H), 5.36 (d, J = 11.9 Hz, 1H), 5.22 (d, J = 11.9 Hz, 1H), 4.44 – 4.33 (m, 1H), 4.25 (m, 4H), 4.13 (dd, J = 7.5, 4.7 Hz, 1H), 3.99 – 3.87 (m, 2H), 3.84 – 3.61 (m, 10H), 3.48 (s, 1H), 3.01 (m, 1H), 2.79 (m, 1H), 2.61 (m, 4H), 2.38 (m, 2H), 1.63 (m, 4H), 1.36 (m, 2H).

^{13}C{^{1}H} NMR δ 174.2, 174.0, 164.1, 153.6, 153.5, 130.2, 129.3, 129.1,127.0, 125.3, 123.7,120.7, 106.4, 104.8, 73.1, 71.0, 70.6, 70.5, 70.1 69.4, 68.2, 68.1, 66.7, 62.7, 61.8, 61.5, 60.2, 55.5, 40.3, 33.8, 28.0, 27.9, 24.7.

ESI-MS, *m/z*: [M-H]$^-$ Calcd for $C_{33}H_{43}N_2O_{12}S^-$ 691.25; Found 691.3.

9b

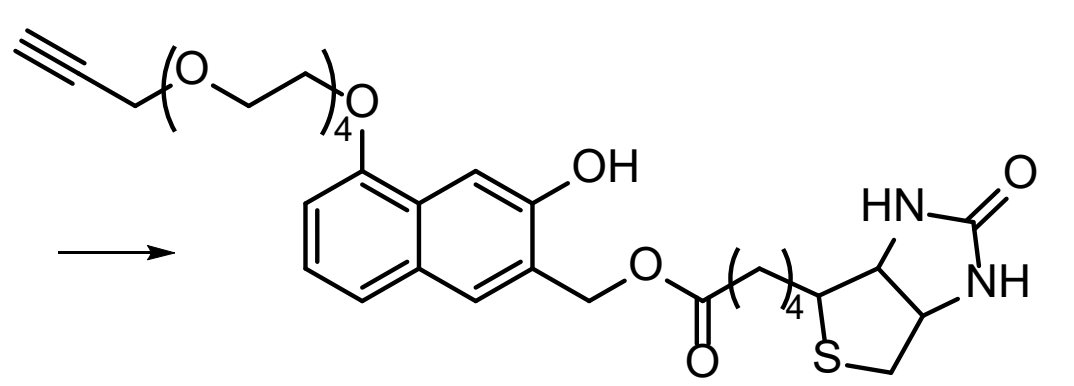

3d

(5-((3,6,9,12-Tetraoxapentadec-14-yn-1-yl)oxy)-3-hydroxynaphthalen-2-yl)methyl (**3d**). A solution of *D*-biotin (0.43 g, 1.75 mmol), EDC (0.50 g, 2.63 mmol), and a catalytic amount of DMAP in 15 mL of anhydrous DMF was added to a solution of **9b** (0.78 g, 1.93 mmol), and stirred overnight. The reaction mixture was separated between 100 mL EtOAc and 100 mL brine, the organic layer was dried and concentrated under reduced pressure. The residue was purified by chromatography (10% MeOH in DCM) to give **3d** (0.44 g, 40%) as colorless crystals.

^{1}H NMR δ 9.14 (s, 1H), 7.68 (s, 1H), 7.63 (s, 1H), 7.30 (d, J = 7.7 Hz, 1H), 7.21 – 7.06 (t, J = 8.4 Hz, 1H), 6.72 (d, J = 7.6 Hz, 1H), 6.15 (s, 1H), 5.73 (s, 1H), 5.38 – 5.19 (m, 2H), 4.41 – 4.27 (m, 1H), 4.21 (m, 2H), 4.14 (m, 2H), 4.09 (m, 1H), 3.98 – 3.87 (m, 2H), 3.84 – 3.75 (m, 2H), 3.72 – 3.57 (m, 10H), 3.43 (s, 4H), 3.12 (s, 1H), 3.02 (m, 1H), 2.68 (m, 1H), 2.65 – 2.52 (m, 1H), 2.43 (m, 1H), 2.40 – 2.31 (m, 2H), 1.70 – 1.43 (m, 4H), 1.45 – 1.27 (m, 2H).

9b → 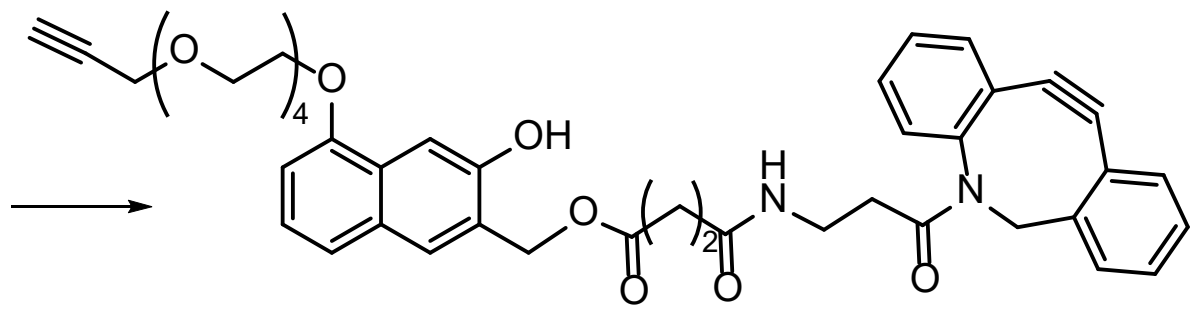 **3f**

PLL 3f. EDC (0.7 g, 3.6 mmol) and catalytic amount of DMAP were added to a solution of ADIBO-ACID (**15**, 0.91 g, 2.4 mmol) in 20 mL anhydrous DMF, the mixture was stirred 15 min., and a solution of **9b** (0.98 g, 2.4 mmol) in 5mL anhydrous DMF was added dropwise. The reaction was stirred overnight and extracted with EtOAc. The organic layer was washed twice with brine, dried, the solvent removed under reduced pressure, and the residue purified by chromatography (EtOAc) to produce **3f** (0.85 g 46%) as a yellow solid.

^{1}H NMR δ 8.08 (s, 1H), 7.69 – 7.60 (m, 3H), 7.35 – 7.11 (m, 9H), 7.13 – 7.03 (m, 1H), 6.71 (d, *J* = 7.5 Hz, 1H), 6.20 (t, *J* = 5.8 Hz, 1H), 5.23 (s, 2H), 5.07 (d, *J* = 13.9 Hz, 1H), 4.26 – 4.15 (m, 2H), 4.07 (dd, *J* = 19.9, 4.7 Hz, 2H), 3.87 – 3.78 (m, 2H), 3.73 – 3.52 (m, 14H), 3.29 (ddd, *J* = 13.5, 10.7, 6.9 Hz, 1H), 3.12 (dt, *J* = 13.4, 8.9 Hz, 1H), 2.68 – 2.19 (m, 8H), 1.88 (ddd, *J* = 16.6, 7.0, 3.6 Hz, 1H).

^{13}C{^{1}H} NMR δ 173.2, 172.5, 171.2, 153.4, 152.8, 151.0, 147.9, 132.2, 129.7, 129.4, 129.1, 128.7, 128.5, 128.4, 127.9, 127.6, 127.2, 125.7, 124.9, 123.3, 122.9, 122.5, 120.7, 114.8, 107.8, 106.6, 105.6, 79.6, 74.7, 71.0, 70.8, 70.6, 70.6, 70.4, 69.7, 69.1, 68.5, 63.1, 58.4, 55.6, 35.3, 34.7, 31.1, 29.9.

ESI MS, *m/z*: $[M+H]^+$ Calcd for $C_{44}H_{47}N_2O_{10}^+$ 763.32; Found 763.4

---

[1] The emission spectra of the lamps and irradiance of the photoreactor with 8 lamps can be found on the manufacturer website: https://luzchem.com/pages/es-lzc-and-ccp-lamps.

[2] He, Q., et al., Size-controlled synthesis of monodispersed mesoporous silica nano-spheres under a neutral condition. Microporous and Mesoporous Materials, 2009. 117(3): p. 609-616

[3] Cheng, Z. Elias, D. R., Kamat, N. P., Johnston, E. D., Poloukhtine, A., Popik, V. V., Hammer, D. A., and Tsourkas, A. *Bioconjugate Chem.*, **2011**, *22*, 2021.

[4] Arumugam, S.; Popik, V. V. *J. Am. Chem. Soc.* **2011**, *133*, 15730.

[5] Kuzmin, A. Poloukhtine, M. Wolfert, and V. V. Popik *Bioconjugate Chem.* **2010**, *21*, 2076.
